# Small immune effectors coordinate peptidoglycan-derived immunity to regulate intestinal bacteria in shrimp

**DOI:** 10.1371/journal.ppat.1010967

**Published:** 2022-11-23

**Authors:** Ping-Ping Liu, Zhe Wei, Zi-Hua Cheng, Xian-Wei Wang

**Affiliations:** 1 Shandong Provincial Key Laboratory of Animal Cells and Developmental Biology, School of Life Sciences, Shandong University, Qingdao, Shandong, China; 2 State Key Laboratory of Microbial Technology, Shandong University, Qingdao, Shandong, China; 3 Laboratory for Marine Biology and Biotechnology, Qingdao National Laboratory for Marine Science and Technology, Qingdao, Shandong, China; University of Toronto, CANADA

## Abstract

Small antibacterial effectors, including lysozymes, lectins, and antimicrobial peptides, are key regulators of intestinal immunity. However, whether there is coordination among them during regulation is an interesting, but largely unknown, issue. In the present study, we revealed that small effectors synergistically regulate peptidoglycan-derived intestinal immunity in the kuruma shrimp, *Marsupenaeus japonicus*. A C-type lysozyme (LysC) was screened as a responsive factor for the intestine-bacteria interaction. LysC functions to restrict intestinal bacteria, mainly by cleaving *Photobacterium damselae* peptidoglycan to generate muropeptides which are powerful stimulators that induce anti-lipopolysaccharides factor B1 (AlfB1), an effective bactericidal peptide. The muropeptides also induce a C-type lectin (Ctl24), which recognizes peptidoglycan and coats bacteria. By counteracting LysC-mediated muropeptide release and AlfB1’s bactericidal activity, Ctl24 prevents the continuous elimination of intestinal bacteria. Therefore, this study demonstrates a mechanism by which small immune effectors coordinate to achieve intestinal homeostasis, and provides new insights into peptidoglycan-derived intestinal immunity in invertebrates.

## Author summary

Intestinal homeostasis largely determines the healthy status of metazoan animals. Uncovering the mechanism for intestinal homeostasis would be helpful to develop new strategies for disease control. In this study, we demonstrated that small immune effectors, including lysozyme, lectin, and bactericidal peptide, collaborate to regulate shrimp intestinal homeostasis in shrimp. Intestinal bacterial peptidoglycan is digested by a lysozyme into muropeptides, which induce the expression of this lysozyme, a bactericidal peptide and a lectin. The former two effectors are able to restrict intestinal bacteria by generating muropeptides and killing bacteria, respectively, while the latter one counteracts their functions. By coating peptidoglycan, this lectin inhibits lysozyme-mediated muropeptides release, and bactericidal peptide-mediated killing of bacteria. Through controlling, but not eliminating intestinal bacteria, these small effectors together achieve a dynamic balance of intestinal bacteria in shrimp. Therefore, this study emphasized the significance of peptidoglycan as the driving force for intestinal homeostasis, and provides insights into generating a healthy intestinal microbiota by utilizing the synergy among small immune effectors.

## Introduction

All metazoan intestines are colonized by a large number of microorganisms, known as the intestinal microbiota [1]. The microbiota and the host share a complex symbiotic, rather than hostile, relationship. The microbiota contributes greatly to the well-being of the host. It performs an immune protective role by preventing colonization and invasion by pathogenetic bacteria, participates in nutrient absorption by producing essential nutrients and facilitating food digestion, and regulates organ formation and tissue regeneration by promoting stem cell proliferation [2]. Certain genetic or environmental factors may break this homeostasis, leading to an imbalance of intestinal microbiota composition and abundance [3]. Dysbiosis will possibly promote certain opportunistic pathogens, markedly increasing the risk of pathogen infection, and threatening the health and even the life of the host. Indeed, many intestinal microorganisms are a potential threat to the host. Therefore, intestinal homeostasis should be tightly regulated.

Small antibacterial effectors play important roles in regulating intestinal homeostasis. Antimicrobial peptides (AMPs) are fundamental components of innate immunity being present in plants to animals, which interact with bacterial membranes or cell walls and destroy the bacterial integrity [4]. Lysozymes can degrade the peptidoglycan (PGN) of bacterial cell wall, and exhibits direct antimicrobial ability [5]. In *Drosophila*, a key set of antibacterial effectors, namely AMPs and lysozymes, are expressed in gut to actively regulate the gut microbiota composition and abundance [6]. The mutant flies lacking either 14 AMPs (defensin, 4 cecropins, drosocin, 2 diptericins, 4 attacins, metchniknowin and drosomycin) or four lysozymes (LysB, D, E, P) host a microbiota of higher stochasticity than the wild-type flies do. Loss of AMPs also led to a significant increase in the load of *Acetobacter* species. This observation, together with previous finding that *Drosophila* AMPs mainly target Gram-negative bacteria [7], indicated that the contribution of AMPs in microbiota regulation was strongly related to their antimicrobial activities. In particular, the *Drosophila* cecropin A1 showed a much higher antimicrobial activity towards the gut commensal bacterium *Acetobacteraceae* strain A911 than towards *Gluconobacter* sp. strain G707. Constant overexpression of this AMP and diptericin could significantly alter the commensal community by eliminating the susceptible A911 strain and enriching the resistant G707 strain [8]. Therefore, these findings suggested that AMPs and lysozymes are key regulators of intestinal microbial ecology. However, considering that many intestinal bacteria are harmless but beneficial to their hosts, these antibacterial effectors should be not just killers, but should also act as key gatekeepers [2,9]. Nevertheless, we lack knowledge as to how the immune effectors coordinate to achieve a balanced intestinal immunity.

Shrimp aquaculture is an important economic industry in many costal countries, but is markedly influenced by serious diseases [10]. Many diseases have been shown to be related to intestinal dysbiosis [11]. Identification of the factors which are critical for shrimp intestinal homeostasis would be helpful to develop strategies for disease control. In the present study, which aimed to identify and characterize such key factors in shrimp intestinal immunity, we performed RNA sequencing (RNA-seq) screening to obtain the candidate genes whose expression was suppressed when the microbiota was eliminated, but were induced upon external pathogenic bacteria invasion. We found that the expression of a C-type lysozyme (LysC) was significantly responsive to both treatments. The role of LysC in intestinal immunity was revealed. More importantly, we found that a C-type lectin (Ctl24), which counteracts the effects of LysC, is necessary for intestinal homeostasis. The synergy between lysozyme and lectin seems to be a core characteristic of the dynamic balance through coordinating the PGN-derived intestinal immunity. Therefore, this mechanism provides a new insight into the significance of synergy between small immune effectors in intestinal homeostasis, and raises the potential to improve intestinal microbiota and control shrimp disease.

## Results

### LysC is a key factor in shrimp intestinal immunity

**Fig 1:**
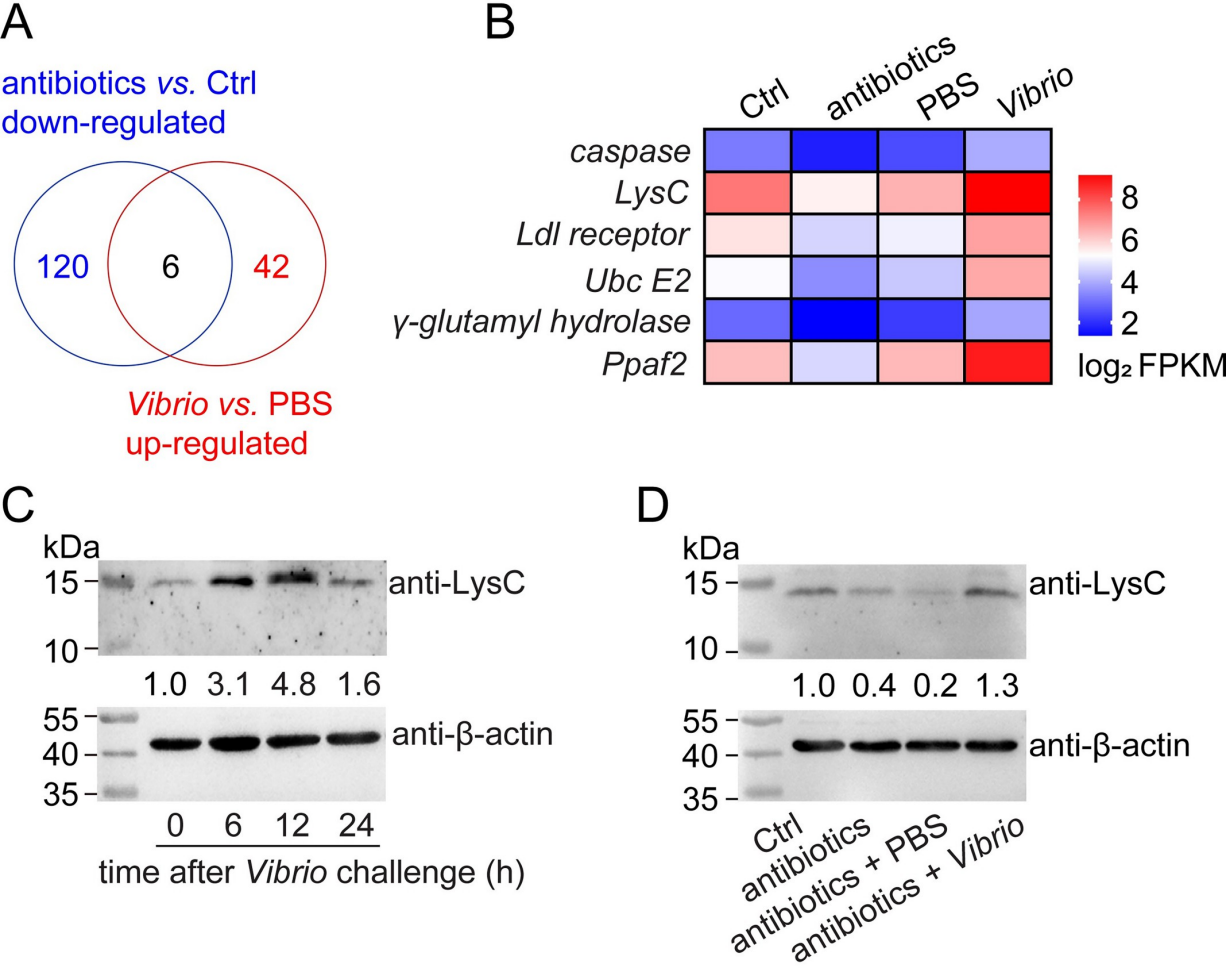
Identification of LysC as a key factor in shrimp intestinal immunity. (A) Venn diagram of the overlap of two sets of differentially expressed genes. Transcriptome sequencing was performed to screen the genes whose expression was induced after *V*. *anguillarum* oral challenge (12 h after challenge) and suppressed after antibiotic feeding (3 d after the treatment). Each group consisted of at least 30 animals. Three biological replicates were performed. Only the genes with an FPKM ≥ 2 in the control group were considered as valid. Differential expression was accepted with a fold change ≥ 2 and an adjusted *p* value ≤ 0.05. (B) Heat map showing the expression level of differentially expressed genes. The mean of the average FPKM value from three biological replicates of each group for each gene were shown after taking the logarithm. LysC, C-type lysozyme; Ldl receptor, low-density lipoprotein receptor; Ubc E2, ubiquitin-conjugating enzyme E2 R2; Ppaf2, phenoloxidase-activating factor 2. (C) Temporal expression profiles of LysC protein after oral challenge. Bacterial suspension (5 × 10^6^ CFU) was introduced orally into the shrimp intestine. LysC protein level in intestine was detected at indicated time after challenge, with β-actin as the internal reference. (D) Expression level of the LysC protein after antibiotic treatment and subsequent *V*. *anguillarum* challenge. The mixture of antibiotics was introduced into shrimp intestine, with water as control. Bacteria were delivered into intestine 3 d later. LysC level was detected 3 d after antibiotics treatment or 12 h after bacterial infection. The western blotting images are representative of three independent replicates. Gray values were analyzed using ImageJ software. Each sample originated from at least five shrimp.

To identify the genes critical for intestinal immunity, a *de novo* transcriptomic analysis was used to identify the target genes whose expression is induced after *V*. *anguillarum* oral challenge but suppressed after elimination of intestinal bacteria using antibiotics. Venn diagram analysis of the differentially expressed genes in the two parts generated six candidate genes (Fig 1A & 1B). The consistency between the sequencing and qRT-PCR results supported the reliability of the screening (S1 Fig, S1 Table). Among the six genes, special attention was paid onto *LysC*, encoding a C-type lysozyme, because it was highly expressed and had been shown to regulate the bacterial communities in shrimp hemolymph; however, the detailed regulatory mechanism was unknown [12,13]. Western blotting was applied to further verify the expression profile of LysC. As shown in Fig 1C, the protein level of LysC was upregulated within 24 h after oral infection. Elimination of intestinal bacteria led to downregulation of the LysC level, and this variation was restored by oral administration of external bacteria (Fig 1D). The significant response of LysC expression suggests it may play an essential role in the intestine-bacteria interaction.

### LysC functions in the regulation of the intestinal microbiota

**Fig 2:**
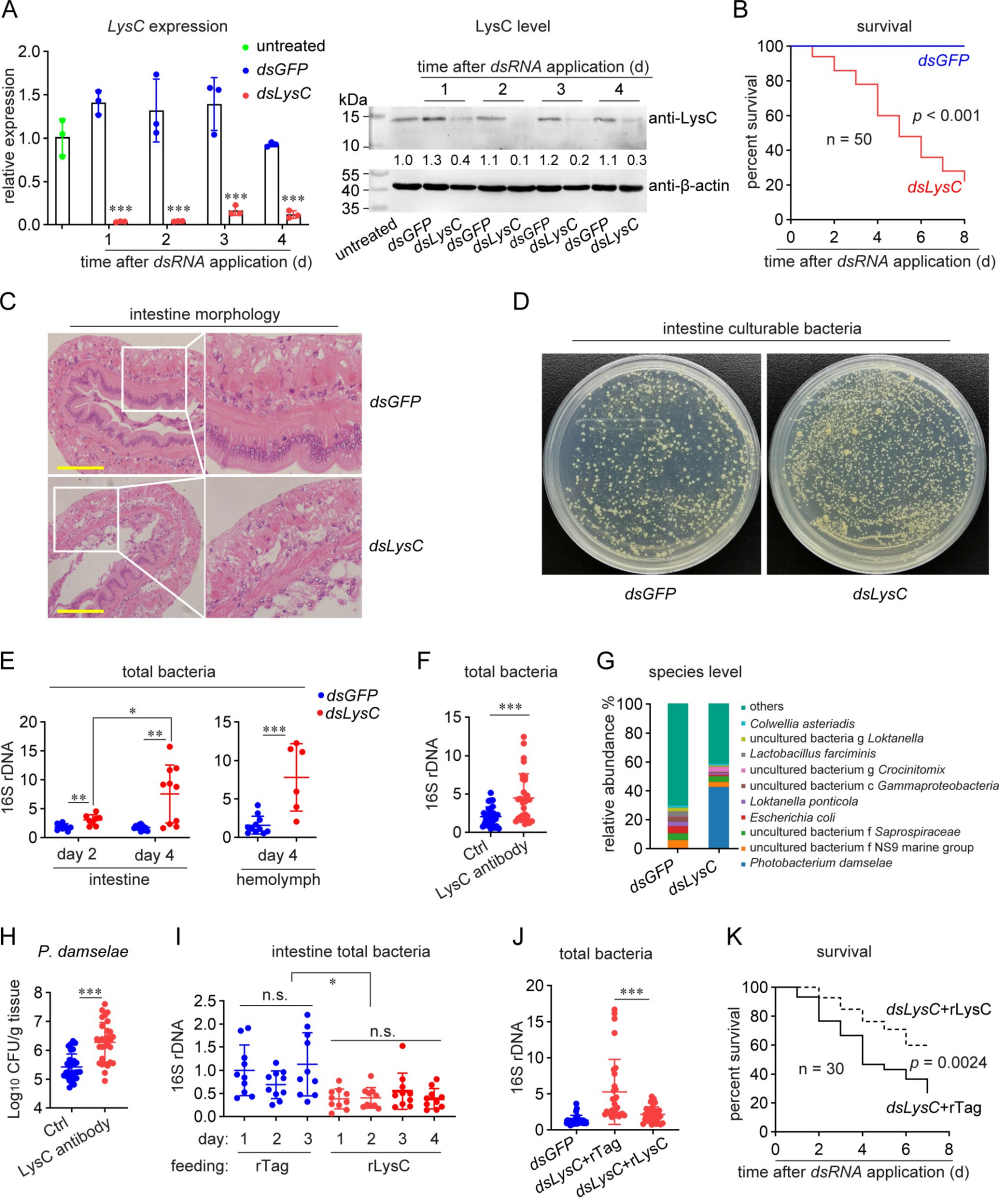
Functions of LysC in maintaining intestinal microbiota homeostasis. (A) RNAi efficiency of *LysC* as determined using qRT-PCR or western blotting. dsRNA was injected into shrimp (5 μg/g body weight). LysC mRNA expression and protein level in intestine was detected at indicated time. Bar chart shows the mean ± SD from three replicates, and the blotting images are representative of three independent replicates. Gray values were analyzed using ImageJ software. Each sample originated from five shrimp. (B) Shrimp mortality analysis after *LysC* knockdown. Shrimp death was recorded each day after *dsLysC* application. (C) Morphological analysis of shrimp intestines after *LysC* knockdown. Intestines were collected at 6 d after dsRNA application, sectioned and stained with H&E. Scale bar = 20 μm. The data are representative of two independent repeats. (D) Influence of the abundance of culturable intestinal bacteria by *LysC* knockdown. Intestines were homogenized at 4 d after dsRNA treatment, and the bacterial load was determined using the plate-counting method. The data are representative of two independent repeats. (E) Influence of the abundance of total bacteria by *LysC* knockdown, as determined using qPCR. Total DNA was extracted after RNAi from the intestine homogenate or hemolymph. The abundance of 16S rDNA was determined using qPCR, and calibrated to host β-actin. (F) Influence of the abundance of total bacteria after LysC neutralization. Native LysC in intestine lumen was neutralized by 5 μg of purified LysC antibody. An antibody which did not recognize any shrimp protein was used as control. The bacterial abundance was determined 3 d later. (G) Variation in the composition of intestinal microbiota after LysC knockdown at the species level, as assessed using 16S high-throughput sequencing. Each sample originated from at least 30 animals. (H) Influence of *P*. *damselae* abundance by LysC neutralization. Total DNA was extracted at 3 d after LysC neutralization to determine *P*. *damselae* abundance by detecting the *ToxR* gene. (I) Influence of total bacterial abundance by rLysC overexpression. The bacterial abundance in intestine was determined by detecting 16S rDNA using qPCR every day after rLysC (2 μg) or control tag administration. (J-K) Rescue of LysC knockdown by rLysC. rLysC or rTag (2 μg) was administered at 24 h after dsRNA application. Total bacterial abundance was determined another 3 d later (J), and the survival rate was recorded (K). For scatter graphs, one dot represents one shrimp. The survival data are representative of two independent repeats. Survival assay was analyzed by log-rank (Mantel–Cox) test, while other statistical analysis was performed using the Students’ t test. *, 0.01 < *p* < 0.05; **, 0.001 < *p* < 0.01; ***, *p* < 0.001.

To reveal the specific function of LysC in intestines, RNAi was applied to knockdown *LysC* expression. Both the qRT-PCR and western blotting results showed that *dsLysC* could significantly reduce the expression level of LysC (Fig 2A). As shown in Fig 2B, *LysC* knockdown led to obvious shrimp death in the absence of external infection, suggesting the probable significance of LysC for shrimp survival. H&E staining results showed that intestinal tissue integrity was damaged in *LysC* knockdown shrimp. *dsLysC*-treated shrimp displayed thinning of the intestinal wall, sloughing of the epithelial cells into the lumen, and a large reduction of microvilli outside the intestinal epithelial cells (Fig 2C). To verify whether the phenotypes were related to an increase of intestinal bacteria, bacterial abundance was detected. The abundance of culturable intestinal bacteria (Fig 2D) and total intestinal bacteria (Fig 2E) were both significantly increased in the *dsLysC* group. Moreover, the abundance at day 4 was higher than that at day 2, suggesting the continuing uncontrolled proliferation of intestinal bacteria after *LysC* knockdown. In addition, an increased bacterial load in shrimp hemolymph was observed. This might be the result of ectopic translocation of increased intestinal bacteria into the hemolymph, or might be due to the uncontrolled proliferation of resident bacteria in hemolymph since LysC can inhibit these bacteria [13]. To confirm the results obtained by using RNAi, anti-LysC antibodies were used to neutralize the native protein, and similar results were obtained (Fig 2F).

To determine whether there is any change of bacterial composition, high throughput 16S rDNA sequencing was performed after *LysC* knockdown. As shown in Fig 2G, at the species level, a strain of *P*. *damselae* was increased. We also determined its abundance using qPCR with specific primers, which confirmed the significant increase of this bacteria after LysC neutralization (Fig 2H). Therefore, we isolated *P*. *damselae* strain from the shrimp intestines for subsequent exploration.

Because LysC is present in intestinal lumen as a soluble protein (S2 Fig), to further confirm its role, rLysC was orally delivered into the intestine to mimics an overexpression like effect. The bacterial abundance in the rLysC group was always significantly lower than that in the control group (Fig 2I). We also administered rLysC into shrimp intestine after *LysC* knockdown, and found that rLysC administration restored the homeostasis in RNAi-shrimp by suppressing the increase of bacterial abundance (Fig 2J), thereby reducing the mortality rate (Fig 2K). Collectively, the above data suggested that LysC is essential for intestinal microbiota homeostasis, the disruption of which would affect shrimp health and survival.

### LysC plays an immunomodulatory role by releasing muropeptides

**Fig 3:**
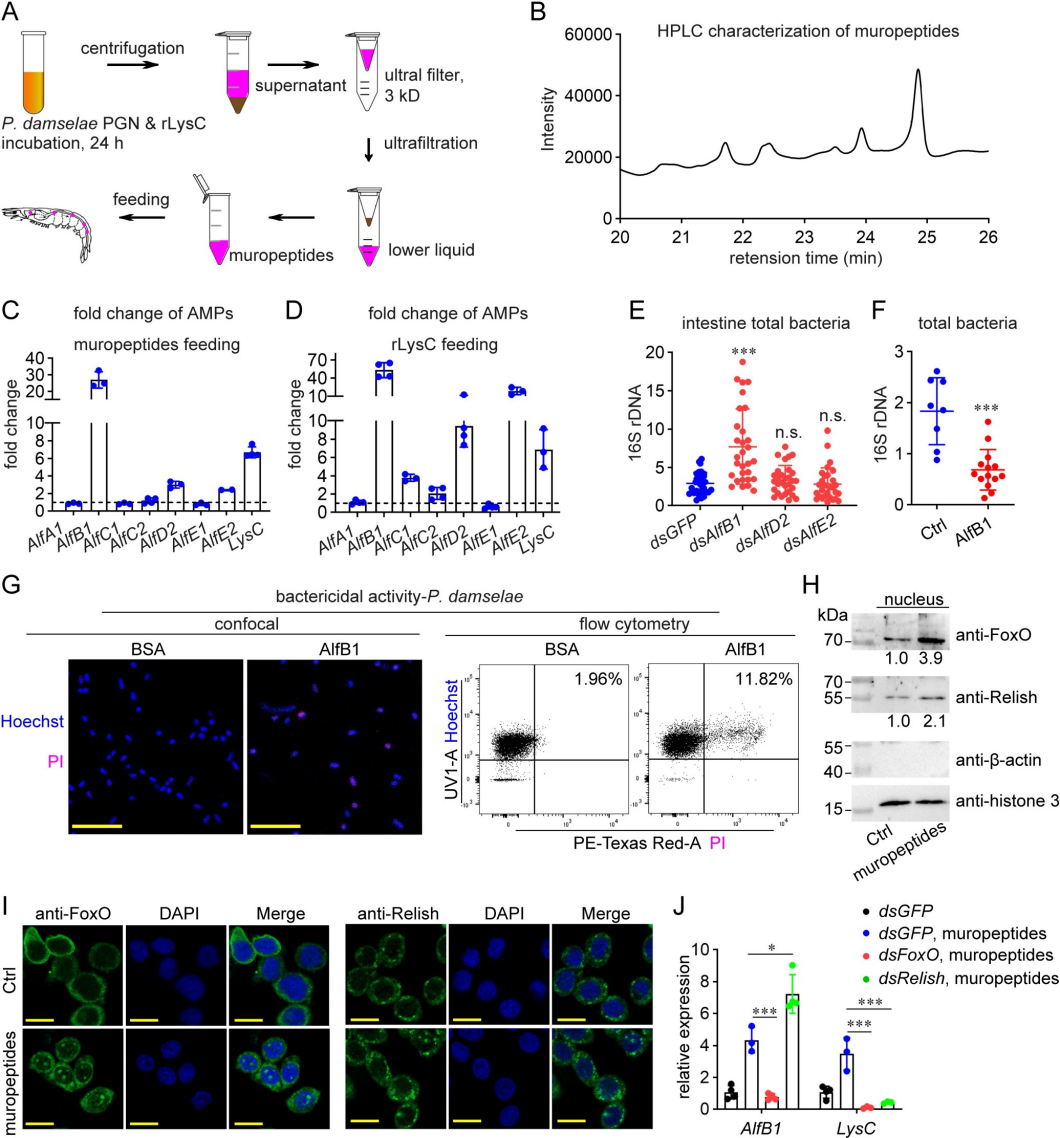
Immunomodulatory role of LysC in intestinal immunity. (A) Schematic illustration of the generation of muropeptides. (B) HPLC characterization of LysC-generated muropeptides. Muropeptide solution (20 μl) was characterized by the reversed-phase column. UV detection was performed at 206 nm. (C) Induction of AMPs by oral treatment with muropeptides. Muropeptides (10 μg) were introduced into intestines. qRT-PCR was performed to detect gene expression 6 h later. (D) Induction of AMPs by feeding with rLysC. rLysC (5 μg) was introduced into intestines, AMPs expression was detected 12 h later. (E) Influence of the abundance of intestinal total bacteria by knockdown of three *Alfs*. The bacterial abundance was determined by detecting 16S rDNA using qPCR 4 d after RNAi. (F) Influence of the abundance of intestinal total bacteria by AlfB1 oral treatment. AlfB1 (2 μg) was administered into intestines, the bacterial abundance was detected 1 d later. (G) Bactericidal activity of AlfB1, revealed by PI staining. *P*. *damselae* were treated with 5 μM of AlfB1 for 2 h at 28°C, and labeled with Hoechst (staining all cells) and PI (staining dead cells). The cells were observed under a confocal microscopy or analyzed using flow cytometry. Scale bar = 10 μm. Flow cytometry collected 10,000 events. Statistical analysis was performed by calculating Q2/(Q1+Q2) × 100%. (H) Induction of the nucleus level of FoxO and Relish by muropeptides, revealed by western blotting. Muropeptides (5 μg) were introduced into intestines. The protein level in nucleus was detected with indicated antibodies 6 h later. Histone 3 and β-actin were detected as internal references for nuclear proteins and cytoplasmic proteins, respectively. (I) Induction of the nucleus level of FoxO and Relish by muropeptides, revealed by immunocytochemical assay. Muropeptides (2 μg) were injected into shrimp hemocoel. Hemocytes were collected 6 h later for immunocytochemical analysis, and observed under confocal microscopy. Scale bar = 10 μm. (J) Expression of the immune effectors after *FoxO* or *Relish* knockdown and muropeptides challenge. Muropeptides (5 μg) were introduced into intestines at 24 h after dsRNA application. Gene expression was detected another 6 h later. All bar charts show the mean ± SD from three replicates. The images are representative of three repeats. One dot represents one shrimp in the scatter graphs. Statistical analysis was performed using the Students’ t test. *, 0.01 < *p* < 0.05; ***, *p* < 0.001.

To reveal how LysC participates in intestinal immunity, we first detected whether it is a direct antimicrobial effector. As shown in S3 Fig, the relative growth of bacteria treated with rLysC was lower than that of the control bacteria, indicating that LysC might exert a direct antibacterial role. Lysozyme can release soluble fragments from intact PGNs through its amidase activity by degrading the β-1, 4-glycosidic bond between PGN N-acetylglucosamine and N-acetylmuramic acid, and these soluble muropeptides are effective stimulators of immunity [14]. Therefore, whether LysC regulates intestinal bacteria in this indirect manner was investigated. We used rLysC to treat PGNs extracted from *P*. *damselae* and collected the cleavage products for high performance liquid chromatography (HPLC) characterization (Fig 3A). As shown in Fig 3B, according to the retention times reported in a previous study [15], the products consisted mainly of PGN monomers and dimers. Moreover, the chromatogram was similar to that obtained by performing the digestion using a commercial lysozyme (S4 Fig). These muropeptides were orally delivered into shrimp intestines to test their immunomodulatory effect. To date, seven members of the anti-lipopolysaccharides factors (Alfs) family, the major AMPs in shrimp, have been identified in *M*. *japonicus* intestines. The results showed that three of them, *AlfB1*, *AlfD2*, and *AlfE2*, were significantly induced by muropeptide challenge (Fig 3C). We also proved that feeding rLysC could also induce the expression of these three Alfs (Fig 3D). In addition, *LysC* expression was also induced by both treatments.

To investigate the effects of three responsive Alfs on intestinal bacteria, RNAi was performed to knockdown their expression (S5 Fig). The abundance of total intestinal bacteria increased only in the *dsAlfB1* group, but not in the *dsAlfD2* or *dsAlfE2* groups (Fig 3E). To confirm its role, AlfB1 protein was orally delivered into shrimp intestines, after which the total bacterial abundance was significantly lower than that in control group (Fig 3F). Alfs are typical bactericidal AMPs; therefore, we detected the bactericidal activity of AlfB1. PI, which can penetrate the membrane of a dead cell, was used to stain dead *P*. *damselae* cells after AlfB1 treatment. As shown in Fig 3G, confocal microscopy images showed stronger PI fluorescent signals in the AlfB1 group than in the control group. Flow cytometry was then used to quantify the ratio of dead cells. Dead cells accounted for 11.82% of the total cells in the AlfB1 group, but only 1.96% in the control group. These data proved that AlfB1 was indeed a direct antibacterial effector that inhibits the proliferation of intestinal bacteria.

The NF-κB-like transcription factor Relish and FoxO have been proven as two central transcription factors in intestinal immunity of both *Drosophila* and shrimp [16–19]. Therefore, we investigated whether LysC-mediated immunomodulation involves them. As transcription factors, both FoxO and Relish are required to remain in nucleus to regulate gene transcription. As shown in Fig 3H, feeding with LysC-generated muropeptides increased the amount of both FoxO and Relish in nucleus. Immunocytochemistry assay was also performed to visualize the distribution of FoxO and Relish, and confirmed this result (Figs 3I & S6). These data proved the modulation of FoxO and Relish activity by LysC-generated muropeptides.

Analysis of the promoter sequences of *AlfB1* and *LysC* (hereafter referred to as the immune effectors) suggested the presence of both FoxO and NF-κB binding sites (S7 Fig). Furthermore, the expression of *AlfB1* decreased significantly after *FoxO* knockdown, while the expression of *LysC* decreased after either *FoxO* or *Relish* knockdown (S8 Fig). Therefore, whether the muropeptides induced their expression in a FoxO or Relish-dependent manner was studied. The results showed that the induction of both *AlfB1* and *LysC* by muropeptides was abolished by *FoxO* knockdown, while *Relish* knockdown only abolished the induction of *LysC* (Fig 3J). Thus, these data suggested that LysC-mediated immunomodulation involves FoxO, and Relish might also be necessary for this process. Taken together, above results showed that LysC can regulate intestinal bacteria in an indirect immunomodulatory manner by releasing bacterial muropeptides and inducing PGN-derived immunity, which is reflected in the nucleus location of transcription factors and the induction of bactericidal AMPs.

### C-type lectin is complementary to LysC in intestinal immunity

We have shown that LysC is essential for intestinal immunity by directly suppressing bacterial growth and indirectly generating muropeptides and inducing AMPs. As mentioned above, the LysC-generated muropeptides could induce *LysC* expression (Fig 3C & 3J). Therefore, there should be a continuous suppression or elimination of bacteria by amplifying immune signals. However, the results in Fig 2I showed that application of rLysC did not lead to a continuous decrease in intestinal bacterial abundance. This prompted us to question if another protein plays a complementary role to LysC in regulating intestinal bacteria. Lysozymes mainly bind to and target bacterial PGNs; therefore, we hypothesized that certain members of the C-type lectin family, whose typical feature is interacting with microbial glycans, might coat the bacterial surface and counteract LysC activity [20].

**Fig 4:**
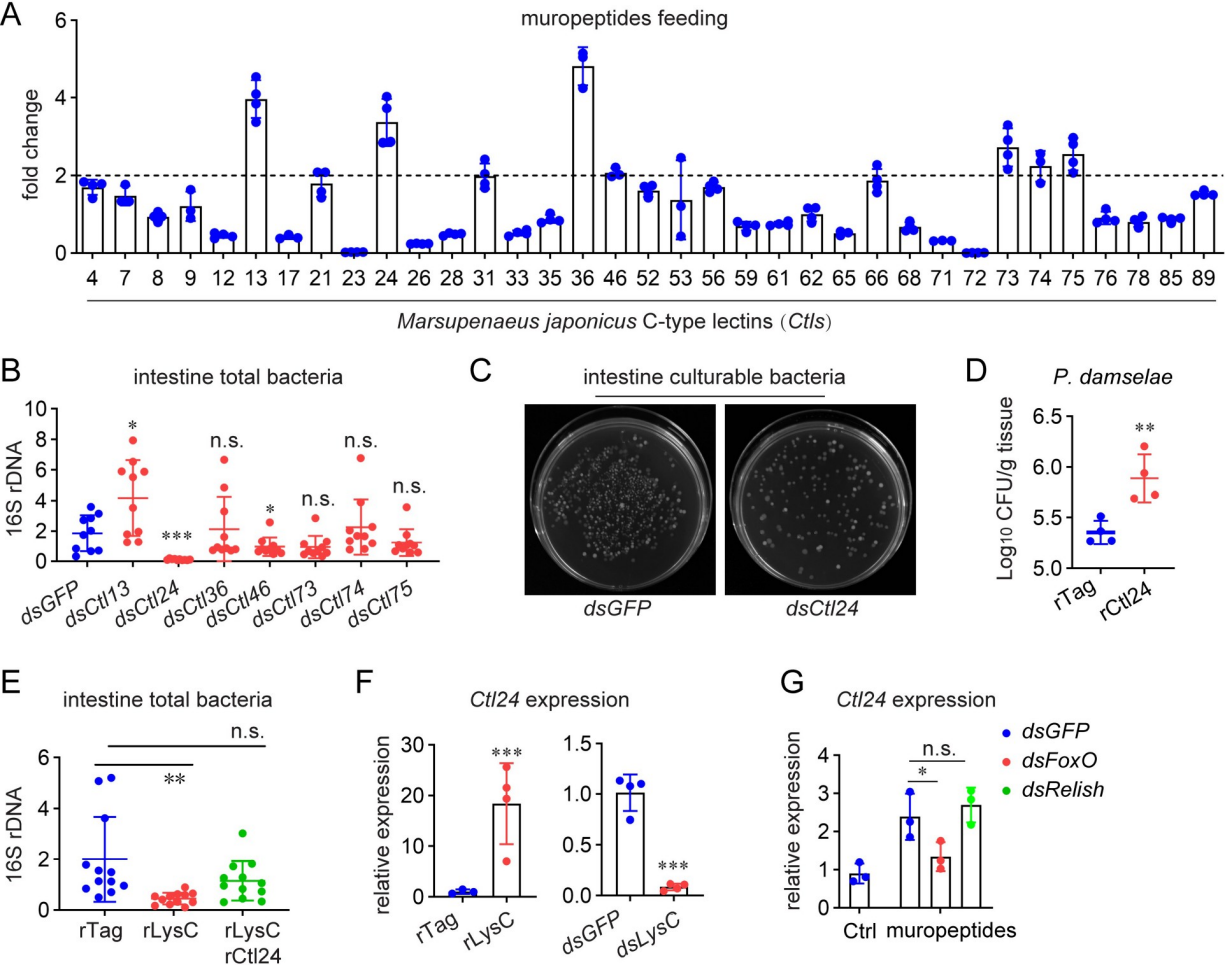
Counteracting role of Ctl24 to LysC. (A) Expression profiles of 34 C-type lectins in the intestines after oral treatment with muropeptides. Muropeptides (10 μg) were introduced into intestines. Gene expression was detected by qRT-PCR 6 h later. (B) Influence of the abundance of intestinal total bacteria by knockdown of responsive *Ctls*. The bacterial abundance was determined by detecting 16S rDNA using qPCR 4 d after RNAi. (C) Influence of the abundance of intestinal total bacteria by *Ctl24* knockdown. The abundance was determined using the plate-counting method. (D) Facilitating colonization of commensal bacteria *P*. *damselae* by rCtl24 in axenic intestines. Antibiotic-treated shrimp were orally delivered with a mixture containing 2 μg of rCtl24 and 1 × 10^5^ CFU of *P*. *damselae*. The bacterial amount was quantified 48 h later by detecting the *ToxR* gene using qPCR. (E) Influence of the abundance of intestinal bacteria by overexpression of rLysC, or rLysC and rCtl24. The bacterial abundance in intestine was determined by detecting 16 S rDNA using qPCR 3 d later protein (2 μg) administration. (F) Regulation of *Ctl24* expression by LysC overexpression or knockdown. *Ctl24* expression in intestine was detected 12 h after rLysC (2 μg) administration, or 24 h after *LysC* RNAi. (G) Expression of *Ctl24* after *FoxO* or *Relish* knockdown and muropeptides challenge. Muropeptides (5μg) were introduced into intestines at 24 h after dsRNA application. Gene expression was detected another 6 h later. All bar charts show the mean ± SD from three replicates. The images are representative of three repeats. One dot represents one shrimp in the scatter graphs. Statistical analysis was performed using the Students’ t test. *, 0.01 < *p* < 0.05; **, 0.001 < *p* < 0.01; ***, *p* < 0.001.

Thirty-four C-type lectins (Ctls) are expressed at a detectable level in shrimp intestines [21]. As shown in Fig 4A, feeding with muropeptides significantly induced the expression of seven Ctls. Subsequently, RNAi of these seven genes was performed to determine if they are involved in intestinal bacterial regulation. The results showed that *Ctl24* knockdown caused a significant decrease in the abundance of total and culturable intestinal bacteria (Fig 4B & 4C). Moreover, *Ctl24* overexpression was observed to facilitate the colonization of *P*. *damselae* in axenic intestines (Fig 4D). These data suggested a role of Ctl24, which is present in intestinal lumen in soluble form (S2 Fig), in maintaining intestinal bacteria, and its potency to counteract LysC activity. To check whether this is the case, rCtl24 was orally delivered into shrimp intestines together with rLysC. As shown in Fig 4E, rCtl24 application abrogated the decrease in intestinal bacteria caused by LysC overexpression, confirming that Ctl24 plays a complementary role to LysC in intestinal homeostasis.

In addition, *Ctl24* expression was also regulated by LysC. LysC overexpression and knockdown significantly increased and decreased *Ctl24* expression, respectively (Fig 4F). Since *Ctl24* expression was also found suppressed by *FoxO* knockdown (S8 Fig), we detected whether the regulation of *Ctl24* by LysC-meditated immunomodulation involves FoxO and Relish. As shown in Fig 4G, *FoxO* knockdown, but not *Relish* knockdown, abolished the induction of *Ctl24* by muropeptides. Therefore, the expression profile of *Ctl24* suggested a possible and interesting feed-back loop in which muropeptides generated by LysC induce Ctl24, which in turn prevents unlimited amplification of LysC activity.

### Ctl24 counteracts LysC-mediated immunomodulatory effects by preventing the release of muropeptides

**Fig 5:**
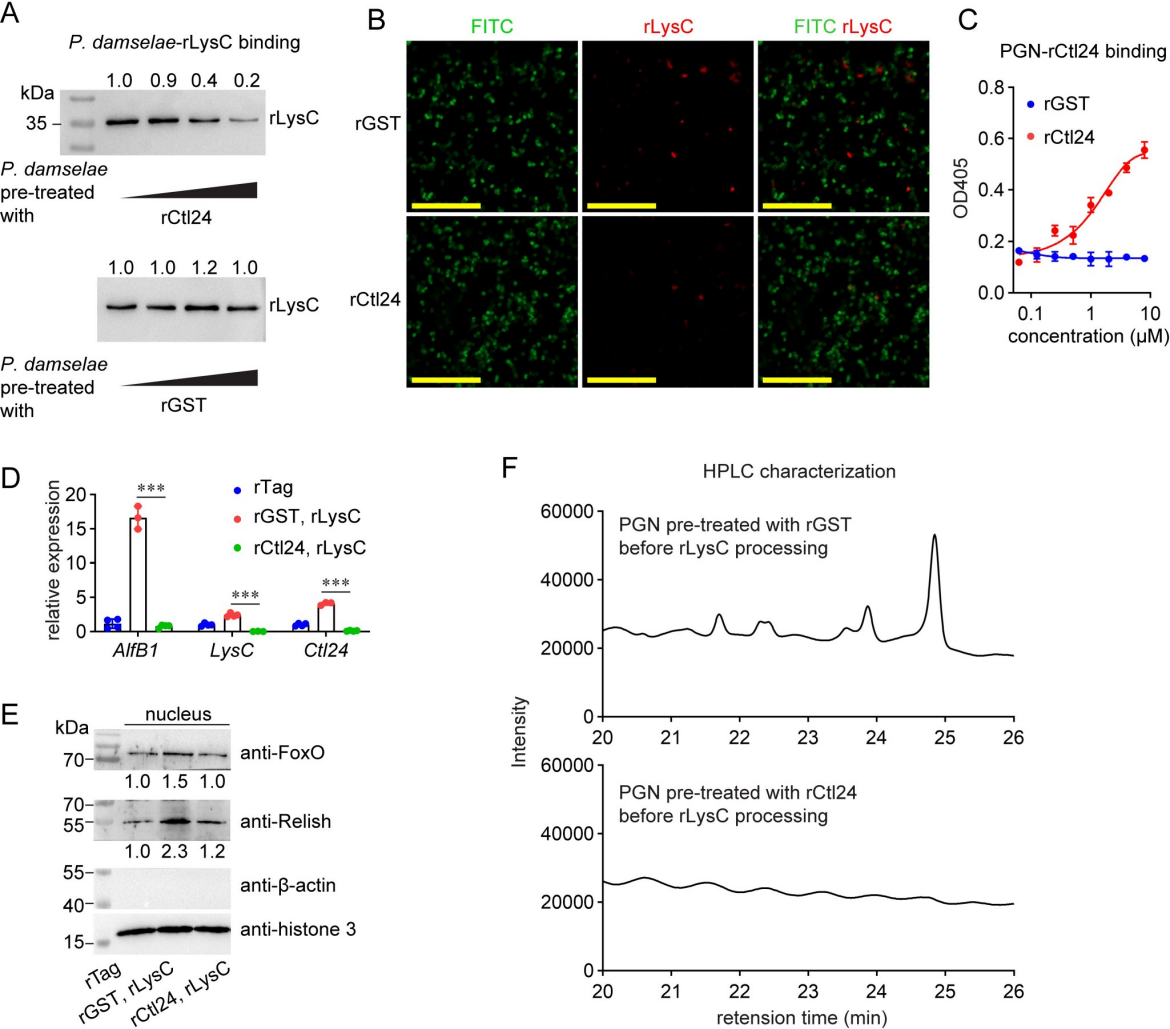
Inhibition of the immunomodulatory role of LysC by Ctl24. (A) Inhibition of *P*. *damselae*-rLysC binding by rCtl24. *P*. *damselae* were pre-treated with rCtl24 or control rGST, and then incubated with rLysC. The amount of rLysC bound to bacterial cells was detected by western blotting using anti-6His antibodies. (B) Inhibition of the deposition of rLysC onto *P*. *damselae* by Ctl24. FITC-labeled bacteria (green) were pre-treated with rCtl24, and then incubated with rLysC. rLysC deposition was visualized by anti-6His antibodies and Dylight 549 Goat Anti-Mouse IgG (red). Scale bar = 20 μm. (C) Binding of *P*. *damselae* PGN by rCtl24. Gradient amount rCtl24 was added into 96-well plates pre-coated by PGN, and the binding of rCtl24 was detected by an ELISA assay. (D-E) Variation of intestinal immunity after feeding with rLysC-generated products when PGN was either or not pre-treated with rCtl24 before rLysC cleavage. *P*. *damselae* PGN was pre-treated with rCTl24 or rGST, and then incubated with rLysC. The digesting products were collected and introduced into intestines. Gene expression (D) and the nucleus level of FoxO and Relish (E) were detected another 6 h later. At least five shrimp were used for a sample. (F) Inhibition of the digestion of PGN by rLysC into muropeptides by rCtl24. *P*. *damselae* PGN was pre-treated with rCTl24 or rGST, and then processed with rLysC. The digesting products were analyzed by HPLC. All bar charts show the mean ± SD from three replicates. The images are representative of three repeats. Statistical analysis was performed using the Students’ t test. ***, *p* < 0.001.

To reveal how Ctl24 counteracts LysC activity, we first investigated whether Ctl24 could inhibit the binding of LysC to intestinal bacteria. As shown in S9 Fig, rLysC could bind to various bacteria isolated from shrimp intestines, including *P*. *damselae*. However, when *P*. *damselae* were pre-treated with rCtl24, binding of rLysC decreased gradually along with increased rCtl24 pre-treatment (Fig 5A). In addition, when FITC-labeled bacteria were pre-coated with rCtl24, rLysC-binding decreased and was much lower than that in the control group, as revealed by confocal microscopy (Fig 5B). These results suggested that incubation with rCtl24 directly reduced the deposition of rLysC onto the bacterial surface.

Next, whether Ctl24 interferes PGN processing by LysC was detected. rCtl24 could bind to *P*. *damselae* PGN in a concentration-dependent manner (Fig 5C). Therefore, rCtl24 was used to pre-treat *P*. *damselae* PGN, which was then subjected to LysC cleavage. The cleavage products were collected and orally delivered into shrimp intestines to determine whether Ctl24 could influence the LysC-mediated immunomodulatory effect. As shown in Fig 5D, the induction of three effectors caused by LysC-generated muropeptides was significantly suppressed when PGN was pre-coated with Ctl24 before LysC cleavage. Moreover, the induction of FoxO and Relish level in nucleus was also suppressed under this circumstance (Fig 5E). HPLC analysis of the cleavage products confirmed that pre-coating by Ctl24 completely blocked muropeptides generation (Fig 5F). Therefore, these results suggested that Ctl24 counteracts LysC function by binding bacterial PGNs and preventing muropeptides release, thereby impairing the LysC-mediated immunomodulatory effect.

### Ctl24 antagonizes the bactericidal effect of AlfB1

**Fig 6:**
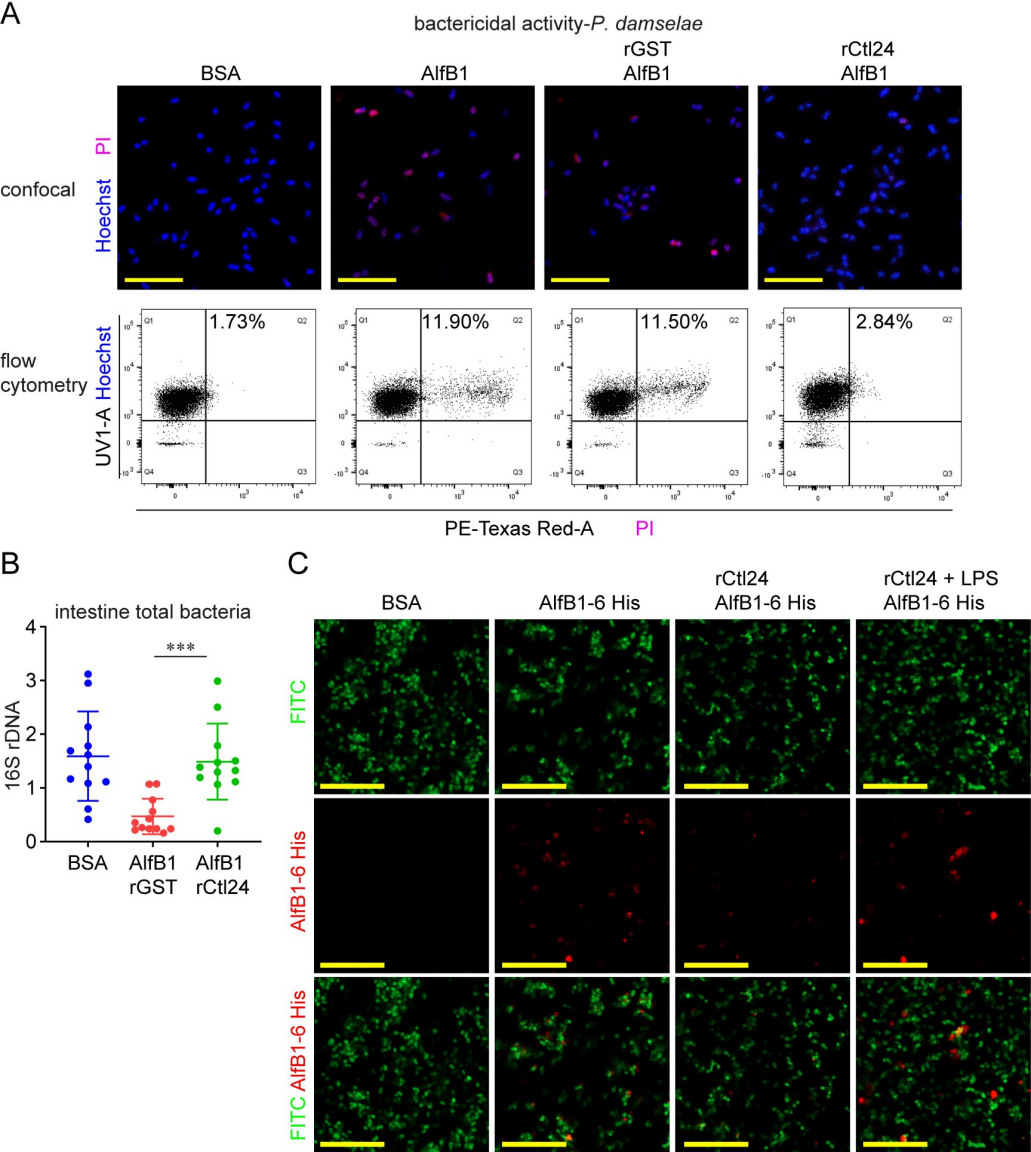
Inhibition of the antimicrobial activity of AlfB1 by Ctl24. (A) Inhibition of bactericidal activity of AlfB1 by Ctl24. rCtl24-pretreated *P*. *damselae* were treated with AlfB1 (5 μM), labeled with Hoechst (staining all cells) and PI (staining dead cells), and observed using confocal microscopy or analyzed by flow cytometry. Scale bar = 10 μm. Flow cytometry collected 10,000 events. Statistical analysis was performed by calculating Q2/(Q1+Q2) × 100%. Data are representative of three repeats. (B) Inhibition of the antimicrobial activity of AlfB1 *in vivo* by rCtl24. Shrimp were treated orally with indicated proteins (2 μg). The abundance of intestine total bacteria was determined by detecting 16S rDNA using qPCR 3 d later. One dot represents three shrimp intestines. Data are representative of two repeats. ***, *p* < 0.001. (C) Inhibition of the deposition of AlfB1 onto *P*. *damselae* by Ctl24. FITC-labeled bacteria (green) were pre-treated with rCtl24 in the presence or absence of LPS, and then incubated with AlfB1-6His. AlfB1 deposition was visualized by anti-6His antibodies and Dylight 549 Goat Anti-Mouse IgG (red). Scale bar = 20 μm. Data are representative of three repeats.

AlfB1 could effectively kill *P*. *damselae*; therefore, we determined whether Ctl24 could influence the bactericidal effect of AlfB1. Confocal microscopy analysis showed that the PI fluorescent signal, representing *P*. *damselae* death caused by AlfB1, decreased when *P*. *damselae* cells were pre-treated with rCtl24. Flow cytometry analysis showed that the number of dead cells accounted for 11.5% in control group, but decreased to only 2.84% in the rCtl24 pre-treatment group (Fig 6A), suggesting that Ctl24 counteracts the bactericidal activity of AlfB1. To determine whether this counteraction is effective *in vivo*, rCtl24 and AlfB1 together were fed into shrimp intestines. This dual overexpression significantly relieved the reduction of intestinal bacterial abundance caused by AlfB1 (Fig 6B). These results proved that Ctl24 could also counteract the bactericidal role of AlfB1, in addition to preventing its continuous expression caused by LysC.

Next, we investigated how Ctl24 antagonizes AlfB1 effect. We detected whether it could prevent the deposition of AlfB1 onto bacterial surface. As shown in Fig 6C, AlfB1 could attach to the surface of *P*. *damselae*. The attachment, however, was blocked when *P*. *damselae* were pre-treated with rCtl24. When LPS, which is also the ligand of rCtl24 (S10 Fig), was used to pre-neutralize the carbohydrates-binding site of rCtl24, this blockage was deprived. These results suggested that Ctl24 may antagonize the bactericidal effect of AlfB1 by preventing its attachment to bacterial surface.

### LysC and Ctl24 coordinate PGN-derived intestinal immunity

**Fig 7:**
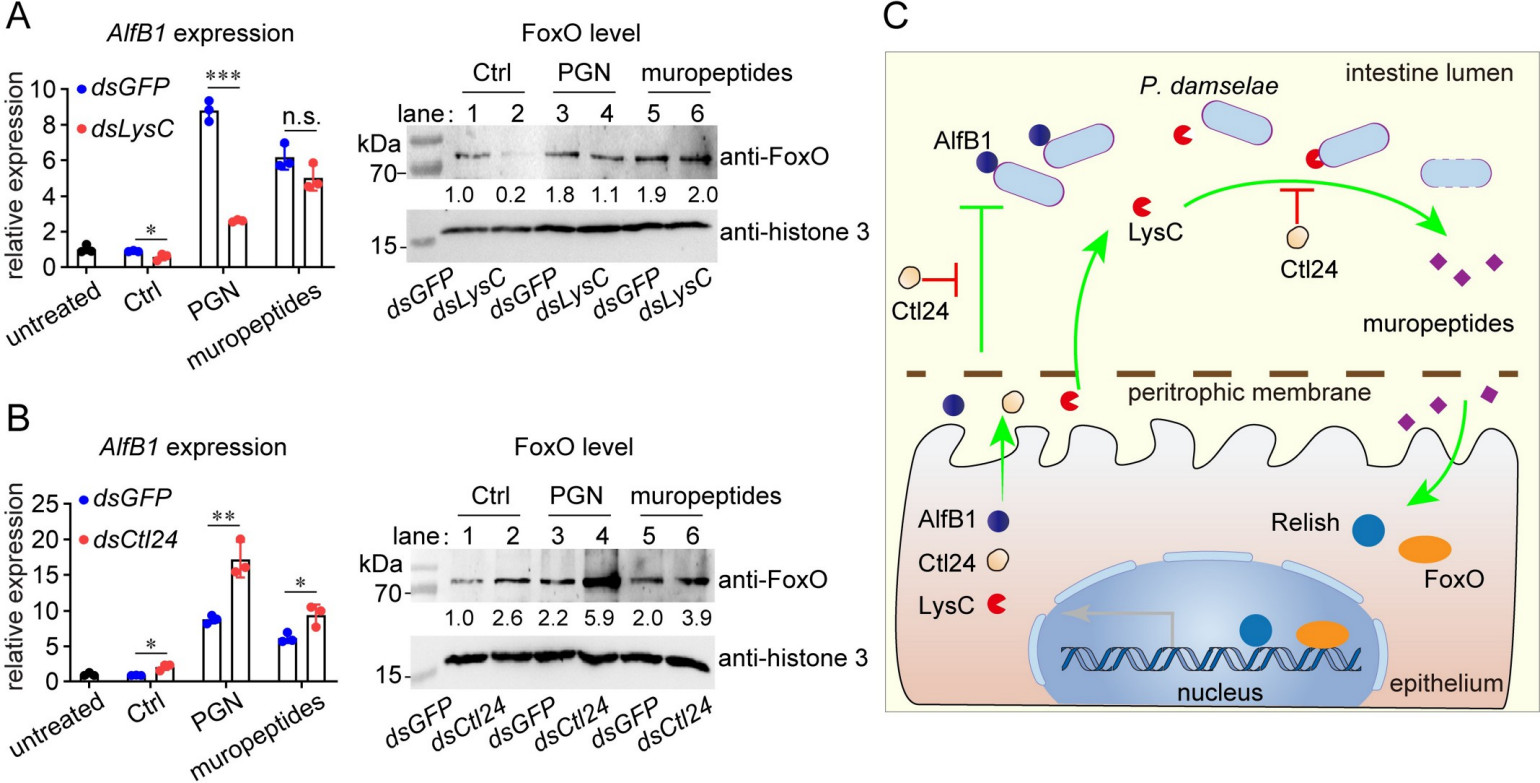
Coordination of LysC and Ctl24 in PGN-derived intestinal immunity. (A) Requirement of LysC for PGN, but not muropeptides, to induce intestinal immunity. PGN or muropeptides (5 μg) were administered into shrimp intestine at 24 h after dsRNA application. *AlfB1* expression (left panel) and FoxO nucleus level (right panel) was detected 6 h later. (B) Requirement of Ctl24 to counteract the intestinal immunity induced by either of PGN and muropeptides. (C) Model of the coordination among immune effectors in intestinal immunity. LysC cleaves intestinal bacterial PGN into muropeptides, which activate both FoxO and Relish to induce the expression of three immune effectors. Coating of bacteria by Ctl24 inhibits the PGN-processing ability of LysC, and inhibits the bactericidal activity of AlfB1. The bar charts show the mean ± SD from three replicates. The images are representative of three repeats. Statistical analysis was performed using the Students’ t test. *, 0.01 < *p* < 0.05; **, 0.001 < *p* < 0.01; ***, *p* < 0.001.

We have shown LysC is essential for intestinal immunity through generating muropeptides, which is effective to induce bactericidal AlfB1. Meanwhile, Ctl24 counteracts this immunomodulatory role of LysC by coating PGN, and avoids the continuous amplification of intestinal immunity. This complementary mechanism is vital for shrimp intestinal homeostasis. To further verify this complementary mechanism, we administered PGN and muropeptides into shrimp intestine after *LysC* or *Ctl24* knockdown to monitor the PGN-derived immunity. As shown in Fig 7A & 7B, *LysC* knockdown and *Ctl24* knockdown decreased and increased *AlfB1* expression, respectively, in a control group without external immune challenge. This might be because, in the normal intestine, less muropeptides were generated after *LysC* knockdown, and more muropeptides were released in the absence of Ctl24 coating. Though *AlfB1* expression was induced by both intact PGN and muropeptides, there was significant difference between two groups. In the PGN group, the results confirmed the role of LysC in releasing muropeptides and the role of Ctl24 in preventing this release. However, when shrimp was administered with muropeptides which do not need further digestion to induce *AlfB1*, *LysC* knockdown did not change the induction, while *Ctl24* knockdown still strengthened the induction. This was probably because Ctl24 could also bind muropeptides to counteract their immunomodulatory activity.

In addition to *AlfB1* expression, we also evaluated the nucleus level of FoxO. As shown in Fig 7A, *LysC* knockdown could reduce the nucleus level of FoxO in the absence of immune challenge (lane 2 *vs* lane 1). Both PGN and muropeptides induced FoxO level (lane 3, 5 *vs* lane 1). LysC knockdown suppressed the induction caused by PGN (lane 4 *vs* lane 3), but did not influence the induction caused by muropeptides (lane 6 *vs* lane 5). This data suggested the requirement of LysC for PGN, but not muropeptides, to induce intestinal immunity. Contrarily, as shown in Fig 7B, *Ctl24* knockdown increased nucleus level of FoxO (lane 2 *vs* lane 1). The induction of FoxO caused by PGN or muropeptides (lane 3, 5 *vs* lane 1) was both enhanced (lane 4 *vs* lane 3, lane 6 *vs* lane 5) after *Ctl24* knockdown. This finding suggested that Ctl24 was able to counteracts the immunomodulatory activities of both intact PGN and muropeptides. Collectively, these data further demonstrated the significance and coordination of LysC and Ctl24 in PGN-derived immunity.

## Discussion

As fundamental components of innate immunity, small antibacterial effectors play important roles in the interaction with bacteria in the intestines. In mammals, high concentration AMPs, RegIII proteins, and lysozymes contribute to building a mucosal barrier against intestinal bacterial infections [22]. In *D*. *melanogaster*, a series of AMPs and lysozymes actively shape the gut microbiota composition and abundance [6]. In the present study, we found that a C-type lysozyme, LysC, is a central effector in intestinal immunity by restricting both the intestinal microbiota and invading bacteria. LysC showed antibacterial activity *in vitro*. More importantly, it plays an immunomodulatory role by cleaving bacterial PGN and generating muropeptides, which are effective stimulators for intestinal immunity. Therefore, LysC functions in intestinal microbiota homeostasis in both a direct and indirect manner. Through inducing AlfB1 with high bactericidal activity, LysC contributes to intestinal antibacterial immunity to suppress excessive proliferation of the intestinal microbiota. By contrast, Ctl24, which is also induced by LysC-mediated immunomodulation, prevents the continuous amplification of intestinal antibacterial immunity by counteracting both the immunomodulatory activity of LysC and the bactericidal activity of AflB1. These three small effectors together achieve a dynamic balance of the host status by controlling, but not eliminating, intestinal bacteria. Nevertheless, LysC is the core molecule in this process.

C-type lectins mainly function in the host-microbiota interaction by binding microbial carbohydrate components [20]. In mammals, RegIIIγ is a bactericidal lectin that leads to pore formation in the bacterial cell membrane via interacting with Gram-positive microbiota PGN and promotes the spatial segregation of the microbiota in intestines [23,24]. In *Aedes aegypti*, galactose-specific C-type lectins (mosGCTLs) facilitate the colonization of commensal bacteria in intestines by preventing the constitutive elimination of the microbiota by AMPs after binding to polysaccharides on the surface of the microorganisms [25]. In shrimp, CTL33 limits the direct contact between the microbiota and the intestinal epithelium by mediating the formation of a microbiota biofilm-like complex [21]. The present study revealed that Ctl24, another C-type lectin expressed in the intestines, contributes to microbiota homeostasis in the intestines by counteracting the effects of a lysozyme to generate a dynamic balance. Therefore, this study highlights the pleiotropic significance of C-type lectins in intestinal homeostasis.

PGN, the characteristic glycans from the bacterial cell surface, is a major driving force for intestinal immunity in both mammals and *Drosophila* [26,27]. In a steady state, PGN fragments are constitutionally liberated from the intestinal bacteria. These muropeptides with different structures, sizes, and activities might be translocated across the intestinal barrier to further affect the host immune response [28,29]. In mammals, certain muropeptides are transported into the host cytoplasm via membrane spanning transporters, and are then recognized by intracellular receptors, including nucleotide binding oligomerization domain containing 1 (NOD1). This recognition initiates the activation of NF-κB, p38, and extracellular signal regulate kinase signaling, ultimately leading to the expression of certain pro-inflammatory cytokines and type I interferon [30,31]. In *Drosophila*, different peptidoglycan recognition proteins (PGRPs) are employed to recognize both extracellular and intracellular PGNs [32]. The membrane associated PGRP-LC receptor recognizes diaminopimelic acid (DAP)-type PGN in both the polymeric form and the monomeric form, leading to activation of the immune deficiency (IMD)-Relish pathway and the production of a range of AMPs [33]. Certain transporters also translocate monomeric PGN into the host cytoplasm, where the tracheal-cytotoxins are recognized by intracellular PGRP-LE [34]. Intracellular sensing also activates the Relish-dependent expression of AMPs [35]. In the present study, we proved that PGN-generated muropeptides are also effective elicitors of the expression of immune effectors in shrimp. This PGN-derived immunity is important for the host to deal with infectious bacteria and intestinal microbiota. However, how these muropeptides are detected to induce the immune effectors is unknown. Analysis of the available genomes of crustacean species have shown that there are no active homologs of *Drosophila* PGRP receptors and mammalian NOD receptors [36]. Therefore, shrimp, and even crustaceans, might use other receptors to recognize PGN and initiate downstream immune signal transduction. The detailed mechanism should be further studied to reveal the likely unique pattern of PGN sensing in crustaceans.

Although the upstream detection mechanism is currently unknown, we showed that two recognized transcription factors in arthropod intestinal immunity, FoxO and Relish, are activated by LysC-generated muropeptides. The significance of Relish in *Drosophila* intestinal epithelial immunity has long been recognized, in which it receives signals from the IMD pathway, acting as transcription factor, and thus regulating the expression of AMPs [37]. By contrast, FoxO functions in intestinal immunity in two ways. First, FoxO can enhance the IMD signaling pathway by promoting the expression of Relish or decreasing the expression of negative regulators of Relish [38]. Second, in the aging gut of *Drosophila*, chronic activation of FoxO reduces the expression of PGRP-SC2, a negative regulator of IMD/Relish. This inhibition results in dysregulation of the Relish activity and causes disordered intestinal symbiosis [39]. FoxO can also directly regulate the expression of antibacterial effector molecules [40]. Although both FoxO and Relish play an indispensable role in intestinal immunity, in this study, we found that FoxO regulates the expression all three effectors, regardless of immune challenge, while Relish regulates LysC expression only. This difference suggested some kind of division of labor between FoxO and Relish. Nevertheless, by evaluating the nucleus level of FoxO and Relish, the important indicator for intestinal immunity, this study emphasized the significance of the synergistic regulation of intestinal immunity by the immune effectors.

In conclusion, this study demonstrated a mechanism by which small immune effectors, including lysozyme, lectin, and AMP, collaborate to regulate shrimp intestinal immunity (Fig 7D). LysC digests intestinal bacterial PGN into muropeptides, which activate both FoxO and Relish to induce the expression of immune effectors. By coating bacteria through interacting with PGN, Ctl24 counteracts the PGN-processing ability of LysC, and antagonizes the bactericidal activity of AlfB1. Through coordinating the PGN-derived immunity, these small immune effectors synergically achieve the intestinal homeostasis, which is vital for host health and growth. Shrimp aquaculture is usually vulnerable to diseases, and many diseases are caused by intestinal dysbiosis [10,11]. Therefore, maintaining and establishing a healthy intestinal microbiota by utilizing the synergy among small immune effectors would be of great significance for shrimp aquaculture. Administration of the recombinant immune effectors together into shrimp intestine may help to generate a stable microbiota, whereas separate administration of a single effector may save an unbalanced microbiota. Moreover, directional breeding concerning the coordination among these immune effectors may favor the tightly-controlled intestinal immunity.

## Materials and methods

### Ethics statement

All animal-related experiments were approved by the Animal Ethical Committee of Shandong University School of Life Sciences (permit number SYDWLL-2021-98).

### Animals and microorganisms

Healthy kuruma shrimp (*Marsupenaeus japonicus*; ~5 g) of normal exterior appearance, behavior and food intake, obtained from an aquaculture farm in Jimo, Shandong, China, were cultured in aerated seawater at 22°C in the laboratory, and fed commercial diets daily. *Vibrio anguillarum* (American Type Culture Collection (ATCC) 43305) and *Photobacterium damselae*, which was isolated from the shrimp intestinal tract, were cultured in Luria–Bertani (LB) liquid medium (3% NaCl, 1% tryptone, 0.5% yeast extract) at 30°C overnight.

### Oral bacterial challenge and antibiotic treatment

*V*. *anguillarum* in the logarithmic growth phase were collected by centrifugation at 8,000 × *g* for 3 min and washed three times using sterile phosphate-buffered saline (PBS) (10 mM Na_2_HPO_4_, 140 mM NaCl, 2.7 mM KCl, and 1.8 mM KH_2_PO_4_, [pH 7.4]). The bacteria were then suspended in PBS and diluted to 1 × 10^8^ colony forming units (CFU)/ml. The bacterial suspension (50 μl) was introduced orally into the shrimp intestine. The control shrimp were treated orally with an equal volume of PBS. For antibiotic treatment, 50 μl of a mixture of antibiotics (ampicillin, 25 mg/ml; kanamycin, 25 mg/ml; streptomycin, 25 mg/ml) was introduced orally into the shrimp intestine, with an equal volume of water as the control. The treatment was repeated 12 h later. Antibiotic-treated shrimp were starved for 48 h after the second antibiotic treatment to allow the host to metabolize the antibiotics. Homogenates of the shrimp intestine tissue in PBS were plated onto 2216E plates (2.4% sea salt, 0.5% tryptone, 0.1% yeast extract, 0.01% FeCl_3_, 1.5% agar powder) and cultured for 24 h at 30°C to verify the effectiveness of the antibiotic treatment. Experiments using the antibiotic-treated shrimp were performed 3 d after the second treatment.

### Transcriptome sequencing

Transcriptome sequencing was performed to identify the genes whose expression was induced after *V*. *anguillarum* oral challenge (12 h after challenge) and suppressed after antibiotic feeding (3 d after the second treatment). Each group contained at least 30 animals. Three independent repeats were performed. Total RNAs from the intestines were extracted using TRIzol Reagent (15596–026, Invitrogen, Carlsbad, CA, USA), and divided into two parts. One part was used for the transcriptome analysis and the other part was preserved for quantitative real-time reverse transcription PCR (qRT-PCR) validation. The commercial transcriptome sequencing was performed by Biomarker Technologies (Beijing, China). The purity, concentration, and integrity of the RNA were determined using the Bioanalyzer 2100 system (Agilent, Santa Clara, CA, USA), and the RNA integrity of each sample was acceptable if the value was above 8.0. Sequencing was performed using the Illumina platform (San Diego, CA, USA). BMKCloud (www.biocloud.net) was applied for bioinformatic analysis. The fragments per kilobase per transcript per million mapped reads (FPKM) method was used to compare gene expression in the different samples. Only a gene with an FPKM ≥ 2 in the control sample was regarded as valid. Differential expression was accepted with a cut-off of 2-fold change, and adjusted *p* value ≤ 0.05. Venn diagram analysis was used for visualization using an online Venn tool (bioinformatics.psb.ugent.be/webtools/Venn).

### Expression profiles analysis

To detect the gene expression profile in intestine, qRT-PCR analysis was performed. Total RNAs were extracted using the TRIzol. The first-strand cDNA was synthesized using a ReverTra Ace qPCR RT Kit (FSQ-101, Toyobo, Osaka, Japan) according to the manufacturer’s instruction, and was used as the template for the quantitative real-time PCR (qPCR). qPCR was performed by using the iQ SYBR Green Supermix (170–8882, Bio-Rad, Hercules, CA, USA) and the CFX96 Real-Time System (Bio-Rad). The PCR program was set as: 95°C for 10 min; 40 cycles at 95°C for 15 s, 60°C for 50 s, and plate reading at 72°C for 2 s; and then a melting period from 65 to 95°C. The data were processed using the 2^−ΔΔCT^ method [41].

For protein level analysis, total intestine proteins were extracted by homogenizing the tissue using Radioimmunoprecipitation assay (RIPA) Lysis Buffer (P0013B, Beyotime, Wuhan, China). The tissue homogenate was centrifuged at 12,000 × *g* for 15 min. The resultant supernatant was collected to determine the protein concentration using a Bradford protein assay kit (C503031, Sangon Biotech, Shanghai, China), mixed with Protein Loading Dye (P0015L, Beyotime) and boiled at 100°C for 10 min for subsequent electrophoresis and western blotting analysis. At least five shrimp were used for each sample.

### Protein recombinant expression and synthesis

The sequences encoding the mature peptides of LysC and Ctl24 were amplified using the specific primers listed in S2 Table, and ligated into plasmids pET32a (+) and pGEX4T-1, respectively. The recombinant vectors were transformed into *Escherichia coli* Rosetta (DE3) and BL21 strains, respectively, for expression under induction by 0.2 mM isopropyl-b-D-thiogalactopyranoside (IPTG) at 28°C for 6 h. The recombinant (r) proteins were expressed as soluble proteins. rLysC was purified using Ni-NTA His Binding resin (70666, Merck, Darmstadt, Germany), and eluted using 250 mM imidazole. rCtl24 was purified using affinity chromatography with ProteinIso GST resin (DP-201, TransGen Biotech, Beijing, China), and eluted using glutathione. The empty vectors were used to generate His-Trx (Thioredoxin) (rTag) and glutathione-S-transferase (GST) (rGST), respectively, as controls. Endotoxins were removed by thorough washing of the column using cold 0.1% Triton X-114 before the final elution [42]. All proteins were dialyzed in PBS, concentrated to 1 mg/ml, and used within 3 days. The peptide corresponding to the active regions of AlfB1 was commercially synthesized (GenScript, Nanjing, China). The amino acid sequence is shown in the S2 Table. For *in vivo* overexpression, rLysC, rCtl24, AlfB1 or the corresponding controls was administered orally into shrimp intestine at a dose of 2 μg. For the phenotype rescue experiment, the oral delivery of protein was performed at 24 h after dsRNA application.

### Antibody generation and purification

rLysC solution (1 mg/ml, 1 ml) and equal volume of complete Freund’s adjuvant (F5881, Sigma-Aldrich, St. Louis, MO, USA) were fully emulsified. The mixture was injected subcutaneously into a New Zealand White rabbit as the first immunization. The second immunization was performed 25 d later, with the complete adjuvant replaced with incomplete adjuvant (F5506, Sigma-Aldrich). The rabbit antiserum was collected 7 d later and stored at -80°C, and were used for western blotting. The specificities of self-made antibodies were shown in S11 Fig. Antisera against FoxO, Relish and β-actin, were generated as described in a previous study [17].

LysC antibodies were purified for in *vivo* administration. Generally, the antiserum was diluted with a buffer (0.15 M NaCl, 20 mM Na_2_HPO_4_, pH 8.0), filtered through a 0.45 μm filter, and applied to a 2 mL Protein A resin column (L00210, GenScript) which was thereafter washed thoroughly with the diluting buffer. After elution with a solution (0.1 M glycine, pH 2.5), the resultant antibodies were neutralized with 1/10 volume of a buffer (1 M Tris-HCl, pH 8.5) immediately, and dialyzed overnight at 4°C in PBS. The concentration of antibody solution was determined by using the Bradford protein assay kit (C503031, Sangon Biotech). An antibody which did not recognize any shrimp protein was purified using the same procedure as control. To neutralize the native LysC in intestine lumen, 5 μg of purified antibodies was orally introduced into the shrimp intestine.

### Western blotting

Western blotting was applied to detect protein levels. 12.5% sodium dodecyl-sulfate polyacrylamide gel electrophoresis (SDS-PAGE) was used to separate protein samples with 100 μg protein loaded per lane. The separated proteins were transferred onto a nitrocellulose membrane using a semidry transfer protocol with a Jim-X Semi-Dry Blotter (Jim-X, Dalian, China). The membranes were blocked with 5% skim milk dissolved in Tris-buffered saline (TBS: 150 mM NaCl, 10 mM Tris-HCl, [pH 8.0]) for 30 min at room temperature, and then incubated with specific primary antibodies for 3 h at room temperature or overnight at 4°C. The membrane was incubated with secondary antibodies for 3 h at room temperature after washing three times with TBST (TBS with Tween-20) and once with TBS. Immunoreactive protein bands were visualized using High-sig enhanced chemiluminescence (ECL) western blotting substrate (180–5001, Tanon, Shanghai, China) and the Tanon 5200 Chemiluminescence Imaging System.

Antibodies against LysC, Relish, and FoxO, were used at 1:200 dilution. β-actin antibodies, anti-Histone H3 polyclonal antibodies (17168-AP-1, ProteinTech, Rosemont, IL, USA) and mouse anti-6His monoclonal antibodies (TA-02, Zhongshan Bio-Tech, Beijing, China) were used at 1:1500 dilution. Horseradish peroxidase (HRP)-conjugated goat anti rabbit antibodies (ZB-2301, Zhongshan Bio-Tech) and HRP-conjugated goat anti mouse antibodies (ZB-2305, Zhongshan Bio-Tech) were used at 1:10,000 dilution in 5% skim milk.

### RNA interference (RNAi)

RNAi was performed to knockdown the gene expression by application of double stranded RNA (dsRNA) *in vivo*. Partial DNA fragments were amplified using specific primers containing a T7 promoter (S2 Table) and used as templates to synthesize dsRNA using a T7 RNAi Transcription Kit (TR102, Vazyme, Nanjing, China). A green fluorescent protein (GFP) DNA fragment was amplified to synthesize *dsGFP* as the control. dsRNA was injected into shrimp at the second abdominal segment using a microsyringe at a dose of 5 μg per gram of body weight. The RNAi efficiency was analyzed using qRT-PCR or western blotting at 24 h post injection. After validating the RNAi efficiency, the subsequent immune challenge assay or phenotype rescue experiments were performed at 24 h after dsRNA application.

### Histological analysis

Histological analysis was performed to detect the impact of *LysC* knockdown on tissue morphology. The intestines were collected and fixed using Davidson’s AFA fixative (30% ethanol, 22% formalin, and 11.5% acetic acid) at 6 d after dsRNA application. After fixing for 24 h, the tissue was dehydrated, embedded in paraffin, sectioned at 7 μm thickness, and stained using hematoxylin and eosin (H&E). The slides were observed under a BX51 microscope (Olympus, Tokyo, Japan) and images were captured using a DP70 digital camera system (Olympus).

### Measurement of the abundance of total and culturable intestinal microbiota

The surface of *M*. *japonicus* was sterilized with 75% ethanol. The midgut was collected and homogenized in 500 μl of sterile PBS. One part of the homogenate was processed to extract midgut total DNA using QIAamp DNA Blood Mini Kit (51104, Qiagen, Hilden, Germany) according to the manufacturer’s instructions. qPCR was performed to detect the 16S rDNA level by using a pair of universal primers (S2 Table). The burden of the intestinal microbiota was expressed as the calibration of 16S rDNA level to host β-actin levels. *P*. *damselae* abundance was quantitatively determined by detecting the *ToxR* gene using specific primers (S2 Table). The other part of homogenate was plated onto a 2216E plate, which was then cultured at 30°C to quantify the abundance of culturable microbiota.

### 16S rDNA high throughput sequencing

Commercial 16S sequencing was performed by Biomarker Technologies. Total microbial DNA was extracted from shrimp intestines using a PowerFecal Pro DNA Kit (51804, Qiagen, Hilden, Germany) according to the manufacturer’s instructions, at 4 d after *dsLysC* injection. At least 30 animals were used to prepare each sample. The quality and quantity of DNA were verified using agarose gel electrophoresis and a Nanodrop ND 2000 spectrophotometer (Thermo Fisher Scientific, Waltham, MA, USA). Sequencing was performed using the PacBio Sequel platform (PacBio, Menlo Park, CA, USA). Effective circular consensus sequence (CCS) data were analyzed using Lima V1.7.0 (https://lima.how/), cutadapt 1.9.1 (https://cutadapt.readthedocs.io/) and UCHIME v4.2 [43] in turn to recognize, filter, and remove chimeras. USEARCH v10.0 [44] software was used to cluster reads at a similarity level of 97.0% to obtain operational taxonomic units (OTUs). SILVA was used as the reference database for taxonomic annotation [45]. Quantitative Insights into Microbial Ecology software (QIIME) software was used to generate species abundance tables at different classification levels [46]. The community structure at the species taxonomic level was displayed using the R language tool (https://www.rproject.org/).

### Muropeptides generation and usage for immune challenge

PGNs were isolated from intestinal bacterium *P*. *damselae* cells as described previously [15]. rLysC was added to the PGN suspension to a final concentration of 0.2 mg/ml. After incubation for 24 h at 37°C with rotation, the mixture was centrifuged at 12,000 × *g* for 20 min to collect the supernatant. To remove rLysC, the supernatant was filtered using a Centrifugal Filter Ultra with a cutoff of 3 kDa (UFC5003, Sigma-Aldrich). After centrifugation at 14,000 × *g* for 20 min, the lower filter contained the muropeptides generated from rLysC cleavage. The control assay was performed with rLysC replaced by equal concentration of rTag. The muropeptides were introduced orally into the shrimp intestines. At specific time points after challenge, intestines were collected to extract total RNA. qRT-PCR was then applied to detect the immune effectors expression.

### HPLC characterization

HPLC was used to analyze the muropeptides (LC-20AT, Shimadzu, Kyoto, Japan), and the UV detector model of this instrument was SPD-M20A, which could realize 190–800 nm full wavelength scanning. Mobile phases included two buffers: buffer A (HPLC-grade water + 0.05% TFA (trifluoroacetic acid)) and buffer B (25% acetonitrile in HPLC-grade water + 0.05% TFA), which were filtered (0.22 μm) and degassed in advance. For chromatographic separation, a Venusil MP C18 (4.6 mm × 250 mm with 5 μm pore size) reversed-phase column (VA952505, Agela, Tianjin, China) was used. HPLC was performed as described previously [15]. Generally, 20 μl of muropeptides solution were injected into the column. The mobile phase comprised 5% buffer B with a flow rate of 1 ml/min for 5min, and then a gradient from 5% to 100% buffer B during 60 min. The column thermostat was set at 40°C. UV detection was carried out at 206 nm.

### Bactericidal activity assay

*P*. *damselae* cells at the logarithmic growth stage were diluted to 10^5^–10^6^ CFU/ml in PBS. The bacteria were pre-incubated with rCtl24 (50 μg/ml) for 2 h or not, and then with 5 μM of AlfB1 for 2 h at 28°C. After three washes, bacteria were stained using 1 μg/ml of propidium iodide (PI) (C0080, Solarbio, Beijing, China) and Hoechst 33258 (B8030, Solarbio) for 15 min at room temperature. After staining, the bacteria were washed three times and suspended in PBS. For flow cytometry, the cell suspension was examined using a flow cytometer (BD FACSAria Fusion, BD, Franklin Lakes, NJ, USA). The data were analyzed using the FlowJo software (FlowJo LLC, Ashland, OR, USA). For confocal imaging, the cell suspension was examined in the multi-track mode using a confocal microscope (LSM 900 with Airyscan, ZEISS, Oberkochen, Germany). The images were analyzed and presented using the ZEN software program (Zeiss).

### Separation of nuclear and cytoplasmic proteins

To determine the distribution of FoxO and Relish, nuclear proteins were extracted using a Nuclear Protein Extraction Kit (R0050, Solarbio), according to the manufacturer’s instructions. Generally, shrimp intestines were homogenized with cytoplasmic protein extraction reagent containing 1 mM phenylmethanesulfonyl fluoride (PMSF). The homogenate was shaken for 20 s and then placed on ice for 3 min. After five times of alternative treatment, the homogenate was centrifuged at 13,000 × *g* for 20 min at 4°C. After three washes using PBS, the sediment was resuspended with nucleoprotein extraction reagent containing 1 mM PMSF, and processed as above. After centrifugation at 13,000 × *g* for 20 min at 4°C, the supernatant was collected as the nuclear proteins. The protein concentration was determined using a Bradford protein assay kit (C503031, Sangon Biotech). At least five shrimp were used for each sample.

### Immunocytochemical analysis

Immunocytochemical assay was performed to detect the distribution of FoxO and Relish. Muropeptides (2 μg) were injected into shrimp hemocoel. At 6 h after injection, the hemolymph was collected into cold anticoagulant (450 mM NaCl, 10 mM KCl, 10 mM EDTA, 100 mM HEPES, [pH 7.45]) containing 4% paraformaldehyde for fixing for 10 min, and centrifuged to collect the hemocytes. The hemocytes were washed with PBS, and spread onto poly-L-lysine coated glass slides. The slides were washed three times with PBS. Thereafter, 0.2% Triton X-100 in PBS was added to the slides for incubation for 10 min. The slides were again washed three times with PBS, and blocked with 3% bovine serum albumin (BSA) in PBS at 37°C for 1 h. FoxO or Relish antibodies (1:100 diluted in 3% BSA) were added to the slides for incubation overnight at 4°C. Goat anti-rabbit Alexa Fluor 488 (A23220, Abbkine, Wuhan, China; 1:1,000 diluted in 3% BSA) was added for incubation for 2 h in the dark. The 4′,6-diamidino-2-phenylindole (DAPI) (AS-83210, AnaSpec, San Jose, CA, USA) was added to stain nuclei for 10 min. Finally, the slides were washed with PBS for observing and capturing with the Zeiss LSM 900 confocal microscope. The images were analyzed and presented using the ZEN software program (Zeiss).

### Bacterial binding assay

*P*. *damselae* was incubated with a gradient concentration of rCtl24 or rGST for 2 h at 28°C. After three washes using TBS, rLysC was incubated with the bacterial cells at a final concentration of 50 μg/ml for 2 h at 28°C. After three washes with TBS, the bacterial cells were suspended with appropriate amount of TBS, mixed with Protein Loading Dye and boiled at 100°C for 10 min. Bound rLysC was analyzed using western blotting using anti-6His antibodies.

For the confocal imaging, fluorescein isothiocyanate (FITC)-labeled *P*. *damselae* was incubated with rCtl24 (50 μg/ml) for 1 h at 28°C. After three washes, the bacteria were incubated with rLysC or AlfB1-6His (50 μg/ml) for 1 h at 28°C. The cells were fixed using 4% paraformaldehyde after three washes. The bacterial cells were smeared onto sialylated slides and stained using the Mouse anti-6His antibody and Dylight 549 Goat Anti-Mouse IgG (A23310, Abcam, Shanghai, China). Subsequently, the bacterial signal was imaged in the multi-track mode using the ZEISS LSM 900 confocal microscope. The images were analyzed and presented using the ZEN software program (Zeiss).

### Carbohydrates binding assay

Binding of rCtl24 to *P*. *damselae* PGN was performed using an enzyme-linked immunosorbent assay (ELISA). Briefly, 50 μl of PGN (80 μg/ml) was used to coat the wells of 96-well plates, followed by air drying at 37°C. The plates were then blocked using 200 μl of 5% BSA in TBS for 2 h at 37°C. After washing four times with TBS, 50 μl of a gradient concentration of rCtl24 was added to the wells. The plates were incubated at room temperature for 3 h. After washing four times with TBS, 100 μl of mouse anti-GST monoclonal antibody (AF5063, Beyotime) (1:2000 diluted with 5% BSA in TBS) was added to each well, and the plates were incubated for 1 h at 37°C. After washing four times with TBS, 100 μl of alkaline peroxidase-conjugated horse anti-mouse secondary antibody (ZB-2310, Zhongshan-Biotech) (1:3000 dilution with 5% BSA in TBS) was added to each well. The plates were incubated for 1 h at 37°C. Finally, 50 μl of p-nitro-phenyl phosphate (1 mg/ml in 10 mM diethanolamine with 0.5 mM MgCl_2_) was added to each well. Incubation was continued for 30 min at 25°C, and the absorbance was measured using a Multiskan FC microplate reader (Thermo Fisher Scientific) at 405 nm. The assays were performed in triplicate independently.

### Quantification and statistical analysis

The density of western blotting bands was quantified using ImageJ software (NIH, Bethesda, MD, USA), and the ratio (tested proteins/internal controls, mean of three replicates) represented the relative expression. The data from the survival assay were analyzed using the log-rank (Mantel–Cox) test using GraphPad Prism 8 software (GraphPad Inc., La Jolla, CA, USA). Other data were analyzed using Students’ t test, and significant difference was accepted at *p* < 0.05.

## Supporting information

S1 Fig Validation of the RNA-seq results using qRT-PCR. The expression of each gene after *V*. *anguillarum* oral challenge (12 h after challenge) and after antibiotic feeding (3 d after the treatment) was detected. Data show mean ± SD from three replicates.
(TIF)

S2 Fig Presence of LysC and Ctl24 in the intestine lumen. Shrimp intestine was perfused using PBS, and the resultant intestinal content was analyzed by western blotting using indicated antibodies. Data are representative of two repeats.
(TIF)

S3 Fig Antibacterial activity of rLysC *in vitro*, analyzed by liquid bacteriostatic experiment. rLysC or the control tag was added to bacterial culture to a final concentration of 10 μM. The incubation was cultured at 25°C. OD600 was detected by a microplate reader after 24 h, and the interval of OD600 reflects bacterial growth. The growth of each bacteria was calibrated to that of the control group (rTag, 0 μM). Data show mean ± SD from three replicates.
(TIF)

S4 Fig Induction of immune effectors by muropeptides generated by commercial lysozyme. (A) HPLC characterization of commercial lysozyme-generated muropeptides. The commercial lysozyme (62970, Sigma-Aldrich) was used to process *P*. *damselae* PGN in the same way to that of rLysC. Muropeptide solution (20 μl) was characterized by the reversed-phase column. UV detection was performed at 206 nm. (B) Induction of immune effectors. Muropeptides (5 μg) generated by commercial lysozyme were introduced into intestines, with water as control. qRT-PCR was performed to detect gene expression 6 h later. The data show the mean ± SD from three replicates. Statistical analysis was performed using the Students’ t test. *, 0.01 < *p* < 0.05. Each sample originated from five shrimp.
(TIF)

S5 Fig RNAi efficiency of muropeptides-induced Alfs. dsRNA was injected into shrimp (5 μg/g body weight). Gene expression in intestine was detected 24 h later. The data show the mean ± SD from three replicates. Statistical analysis was performed using the Students’ t test. *, 0.01 < *p* < 0.05; ***, *p* < 0.001. Each sample originated from five shrimp.
(TIF)

S6 Fig Digitization of the colocalization of FoxO or Relish with nuclei after muropeptides challenge. The Wright Cell Imaging Facility (WCIF) ImageJ software was used to quantify the co-localization in the immunocytochemistry images shown in Fig 3I.
(TIF)

S7 Fig Analysis of the promoters of LysC, Ctl24 and AlfB1. The sequences were obtained from the *M*. *japonicus* genome (GenBank GCA_017312705.1 and GCA_002291165.1), verified by PCR and sequencing. The transcription start sites were determined by integrally comparing and analyzing the cDNA sequence and the transcriptome sequencing dataset. Potential elements were analyzed using the online PROMO 3.0 tool and JASPAR tool.
(TIF)

S8 Fig Regulation of immune effectors by FoxO and Relish in intestine. dsRNA was injected into shrimp (5 μg/g body weight) to knockdown *FoxO* or *Relish* expression. Expression of *AlfB1*, *LysC* and *Ctl24* were detected 24 h later. The data show the mean ± SD from three replicates. Statistical analysis was performed using the Students’ t test. *, 0.01 < *p* < 0.05; ***, 0.001 < *p* < 0.01. Each sample originated from five shrimp.
(TIF)

S9 Fig Binding of rLysC to intestinal bacteria. Bacteria (10^8^ CFU) were incubated with rLysC or the control tag (50 μg/ml) for 2 h. After three washes with TBS, the bacterial pellets were processed, and the bound proteins were detected by western blotting using anti-6His antibodies. Data are representative of two repeats.
(TIF)

S10 Fig Binding of LPS by rCtl24. Gradient amount rLysC was added into 96-well plates pre-coated by LPS (L2880, Sigma-Aldrich; 4 μg/well), and the binding of rCTl24 was detected by an ELISA assay. The data show the mean ± SD from three replicates.
(TIF)

S11 Fig Specificities of LysC and Ctl24 antibodies. Intestine samples were collected 24 h after dsRNA application, and analyzed by western blotting using indicated antibodies.
(TIF)

S1 Table Detailed information of RNA-seq results. (XLSX)

S2 Table Primers and peptides used in this study. (DOCX)
