# Supplementary figures and images for: Small immune effectors coordinate peptidoglycan-derived immunity to regulate intestinal bacteria in shrimp
Source: PLoS Pathog. 2022 Nov 23;18(11):e1010967. doi: 10.1371/journal.ppat.1010967 (PMC9683584; doi:10.1371/journal.ppat.1010967)

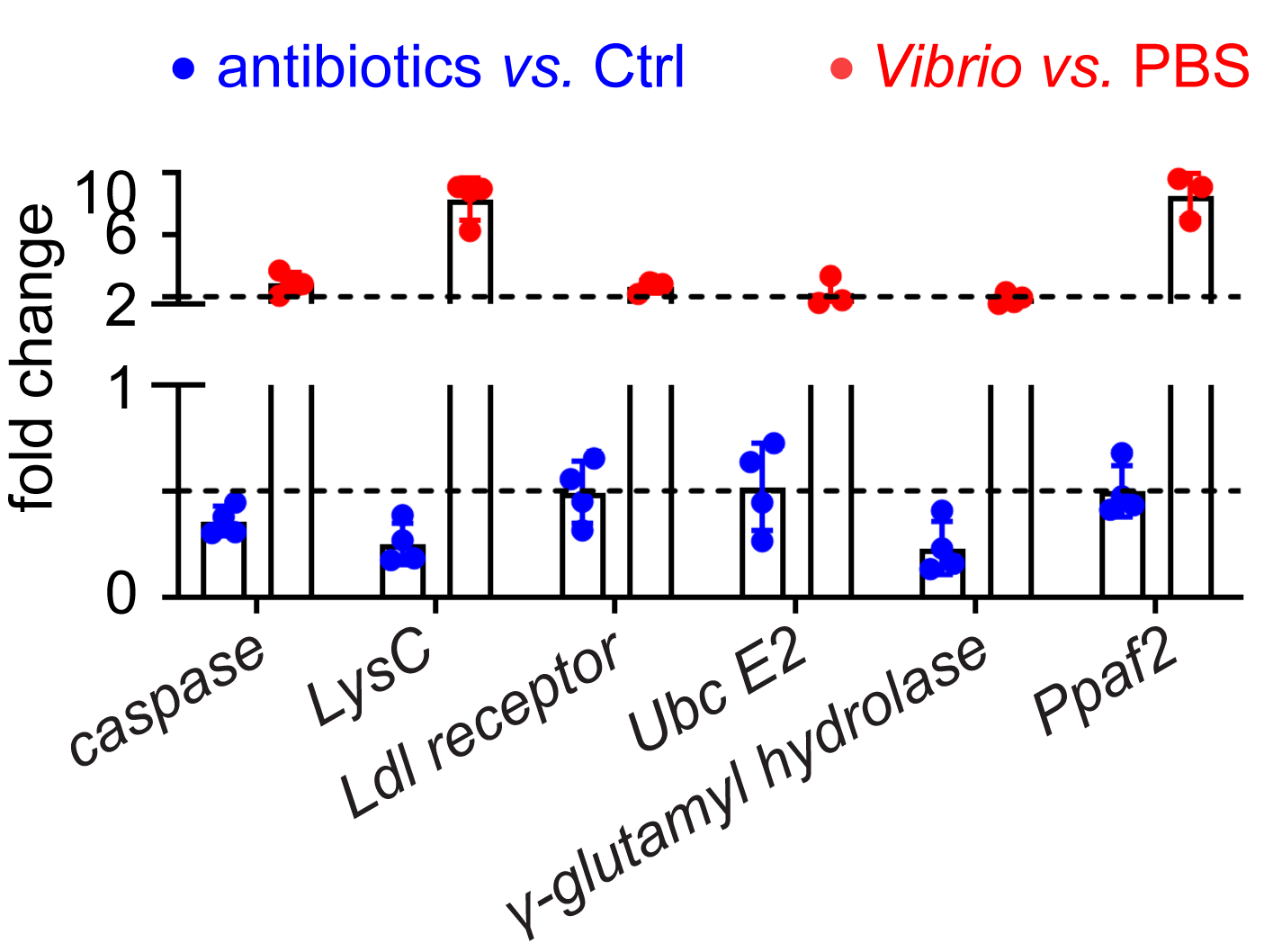

Supplement: S1 Fig — The expression of each gene after V. anguillarum oral challenge (12 h after challenge) and after antibiotic feeding (3 d after the treatment) was detected. Data show mean ± SD from three replicates. (TIF) [file ppat.1010967.s001.tif]

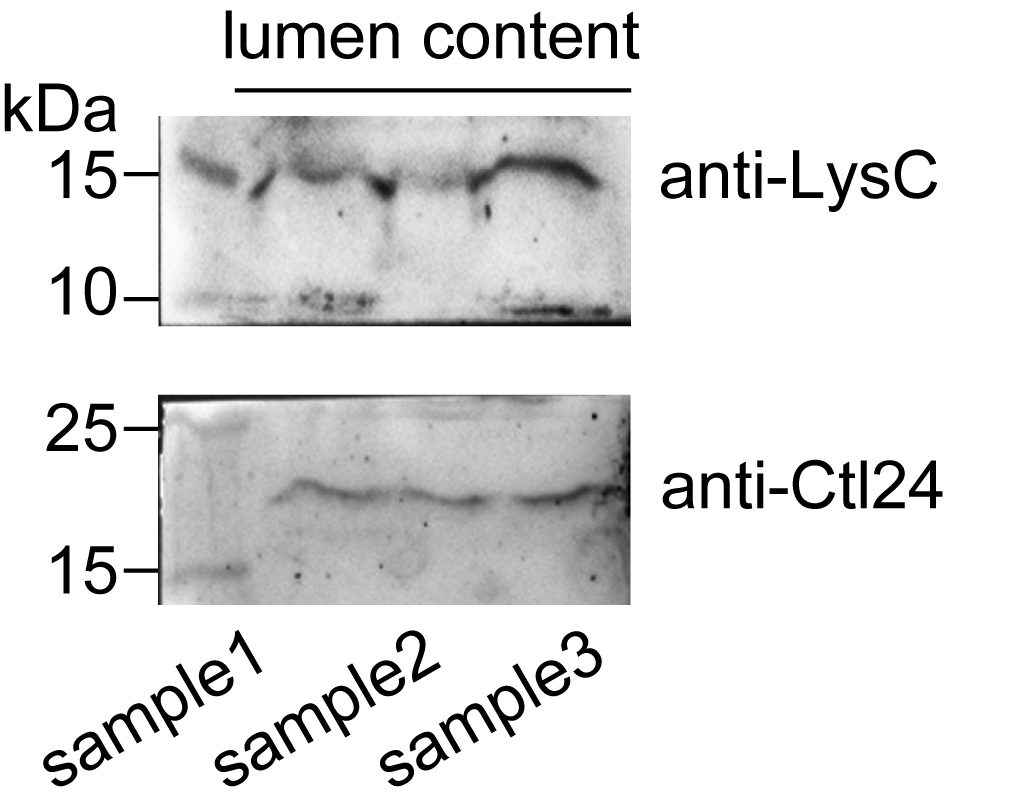

Supplement: S2 Fig — Shrimp intestine was perfused using PBS, and the resultant intestinal content was analyzed by western blotting using indicated antibodies. Data are representative of two repeats. (TIF) [file ppat.1010967.s002.tif]

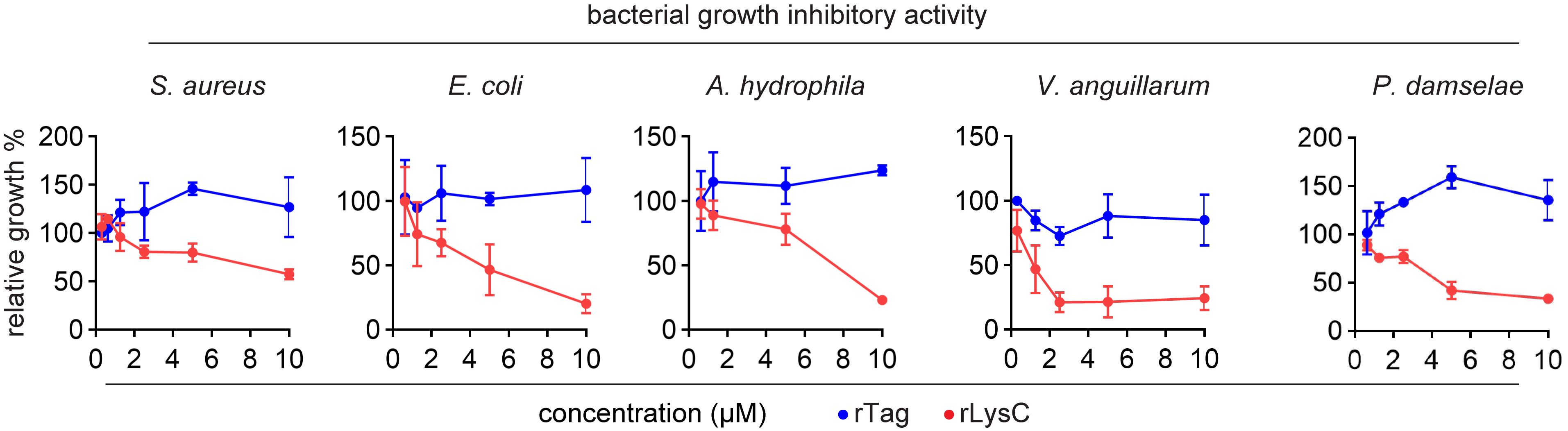

Supplement: S3 Fig — rLysC or the control tag was added to bacterial culture to a final concentration of 10 μM. The incubation was cultured at 25°C. OD600 was detected by a microplate reader after 24 h, and the interval of OD600 reflects bacterial growth. The growth of each bacteria was calibrated to that of the control group (rTag, 0 μM). Data show mean ± SD from three replicates. (TIF) [file ppat.1010967.s003.tif]

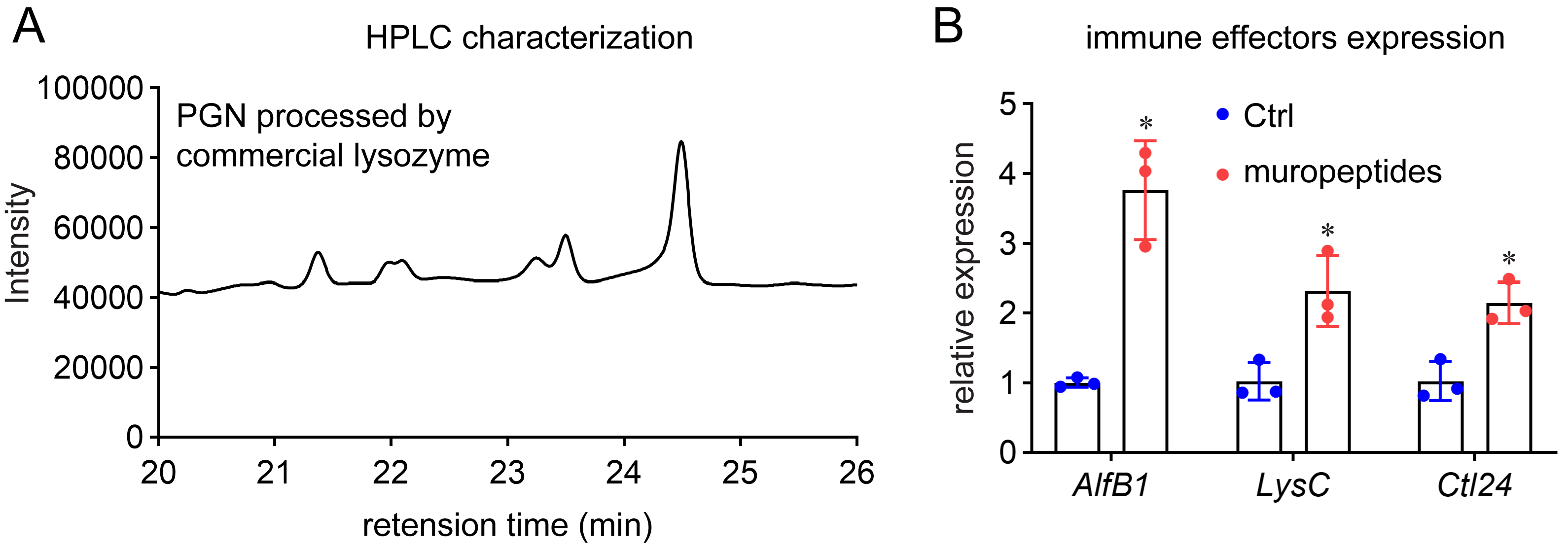

Supplement: S4 Fig — (A) HPLC characterization of commercial lysozyme-generated muropeptides. The commercial lysozyme (62970, Sigma-Aldrich) was used to process P. damselae PGN in the same way to that of rLysC. Muropeptide solution (20 μl) was characterized by the reversed-phase column. UV detection was performed at 206 nm. (B) Induction of immune effectors. Muropeptides (5 μg) generated by commercial lysozyme were introduced into intestines, with water as control. qRT-PCR was performed to detect gene expression 6 h later. The data show the mean ± SD from three replicates. Statistical analysis was performed using the Students’ t test. *, 0.01 < p < 0.05. Each sample originated from five shrimp. (TIF) [file ppat.1010967.s004.tif]

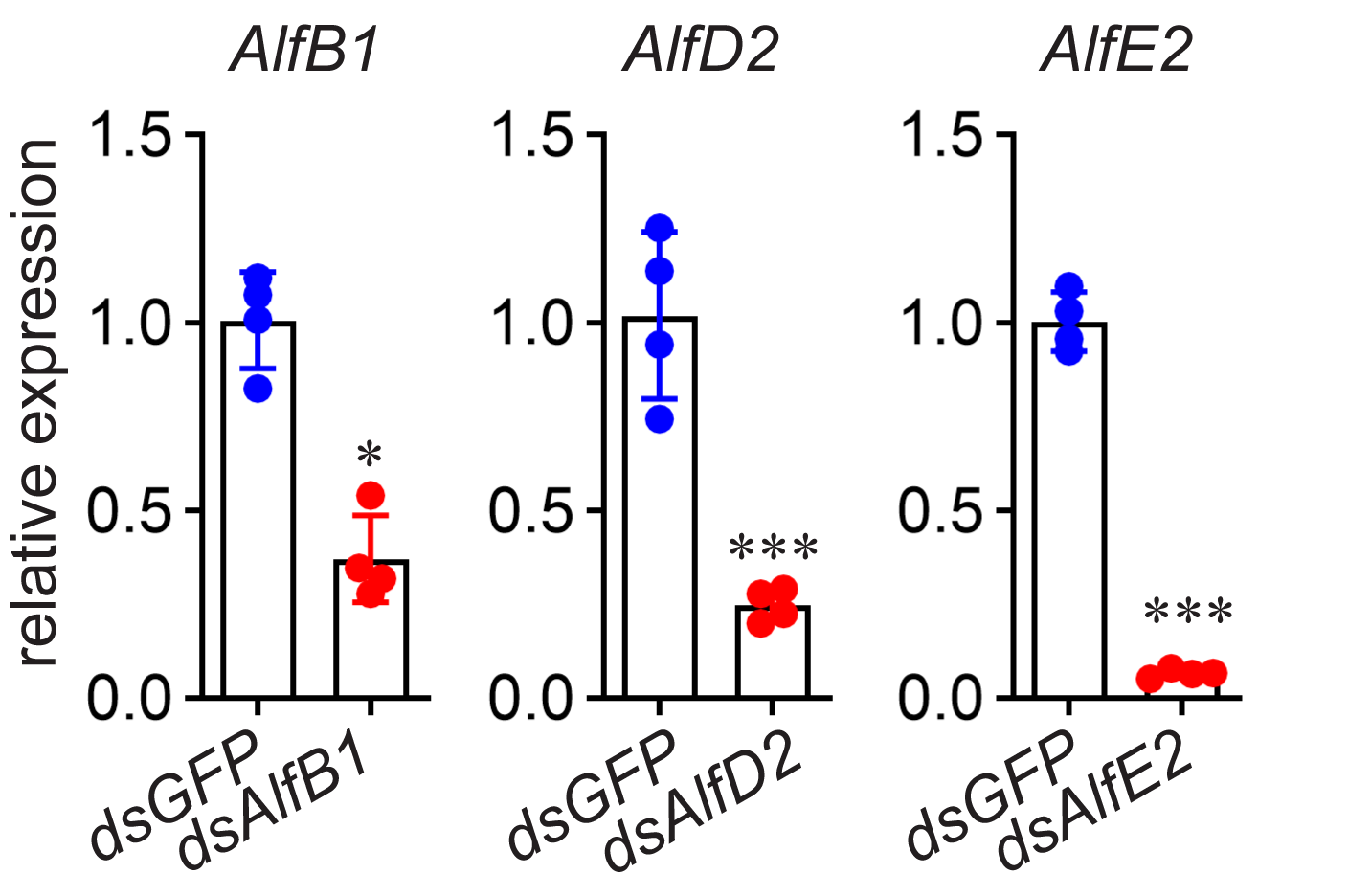

Supplement: S5 Fig — dsRNA was injected into shrimp (5 μg/g body weight). Gene expression in intestine was detected 24 h later. The data show the mean ± SD from three replicates. Statistical analysis was performed using the Students’ t test. *, 0.01 < p < 0.05; ***, p < 0.001. Each sample originated from five shrimp. (TIF) [file ppat.1010967.s005.tif]

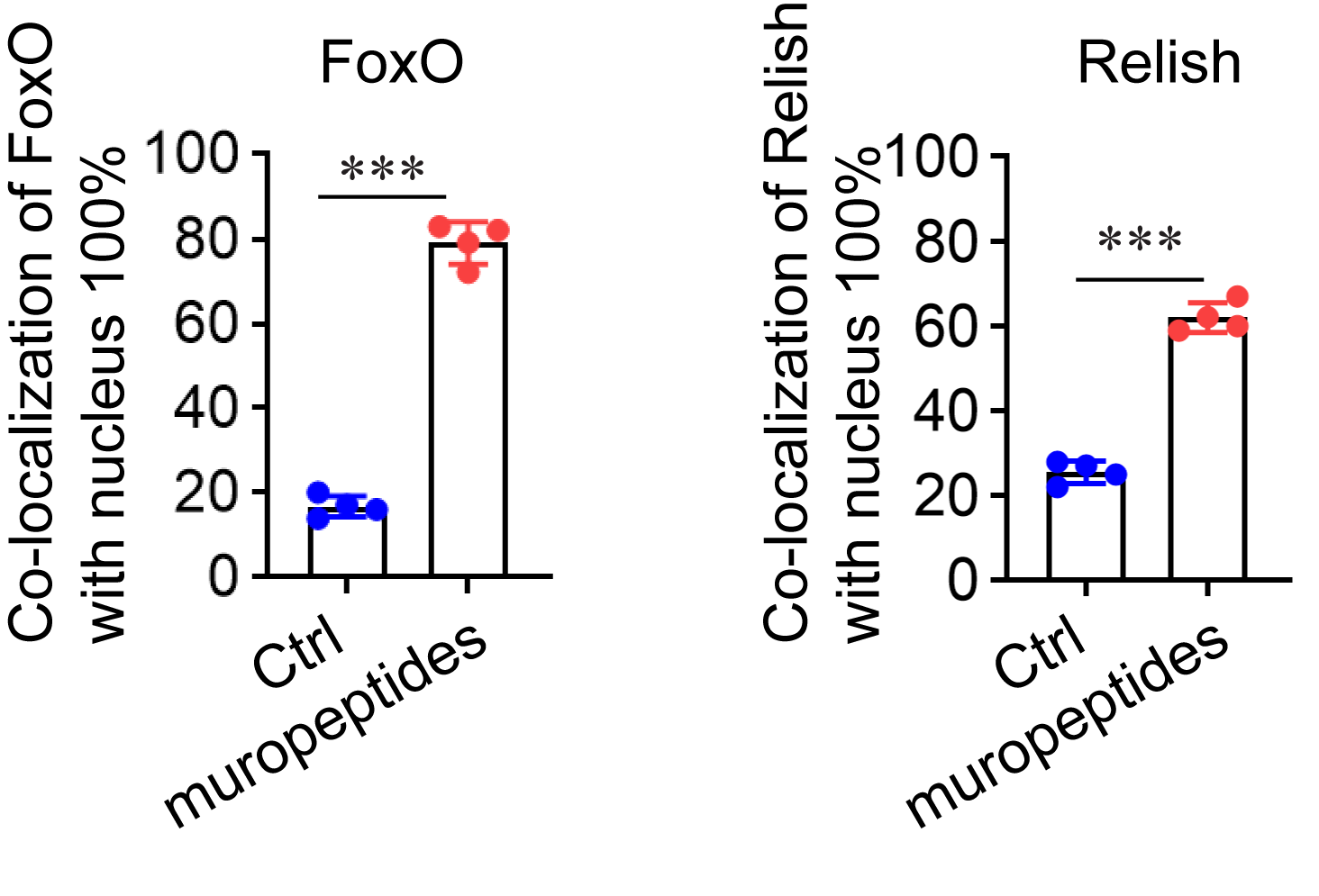

Supplement: S6 Fig — The Wright Cell Imaging Facility (WCIF) ImageJ software was used to quantify the co-localization in the immunocytochemistry images shown in Fig 3I. (TIF) [file ppat.1010967.s006.tif]

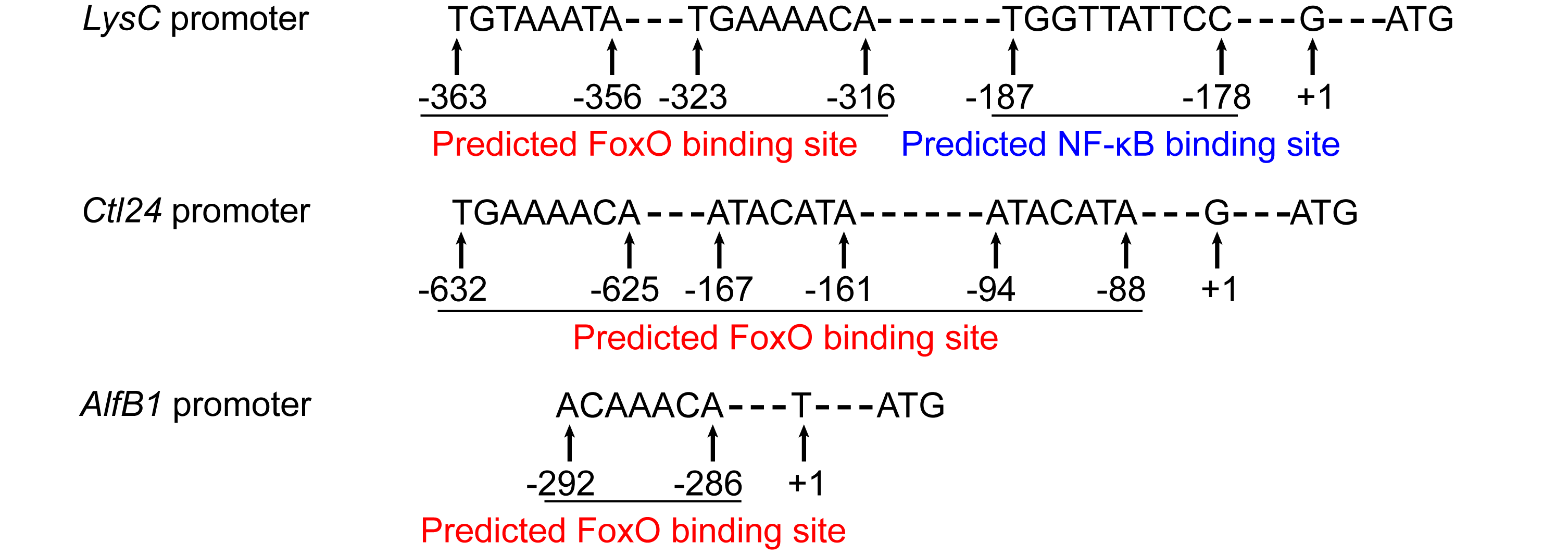

Supplement: S7 Fig — The sequences were obtained from the M. japonicus genome (GenBank GCA_017312705.1 and GCA_002291165.1), verified by PCR and sequencing. The transcription start sites were determined by integrally comparing and analyzing the cDNA sequence and the transcriptome sequencing dataset. Potential elements were analyzed using the online PROMO 3.0 tool and JASPAR tool. (TIF) [file ppat.1010967.s007.tif]

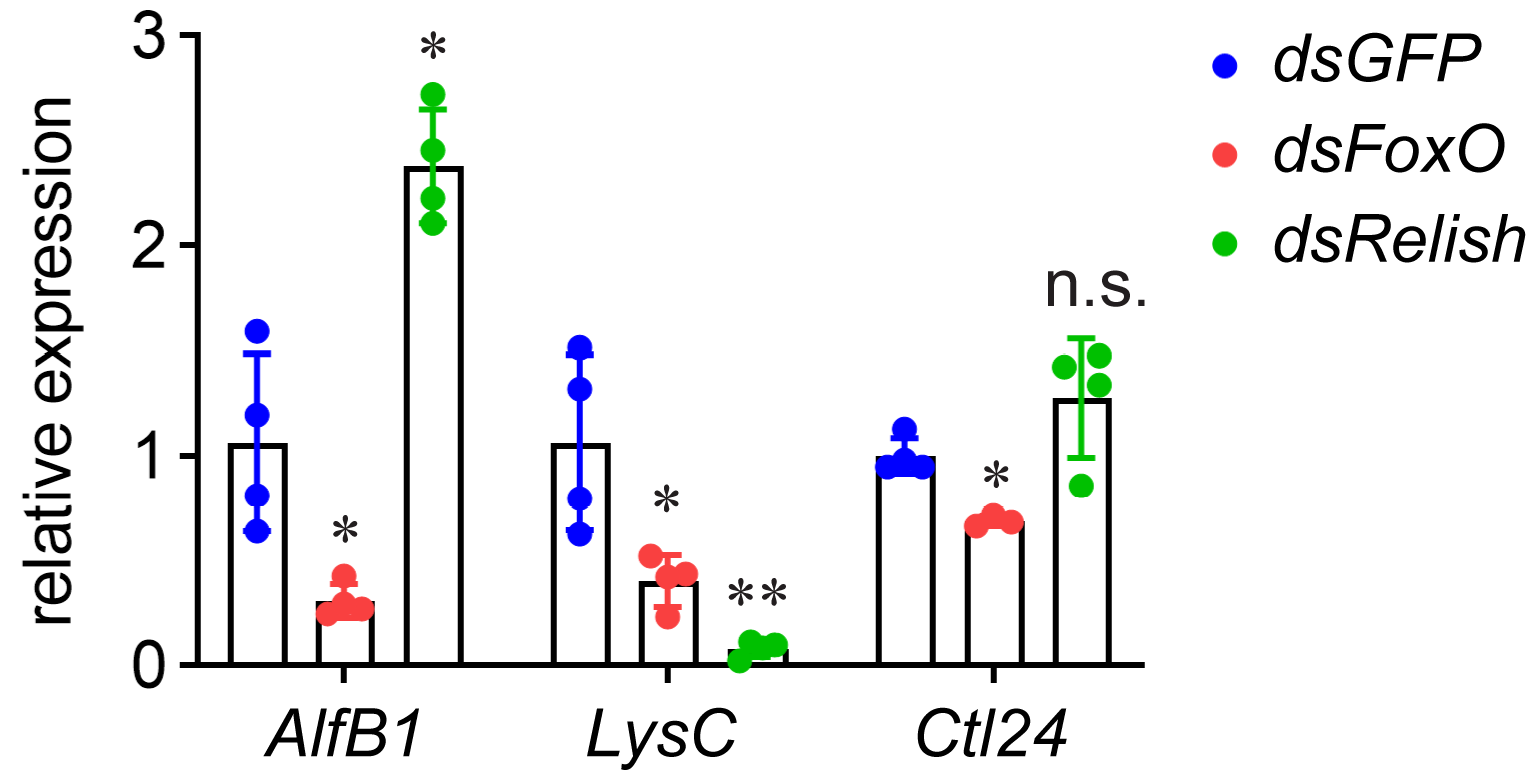

Supplement: S8 Fig — dsRNA was injected into shrimp (5 μg/g body weight) to knockdown FoxO or Relish expression. Expression of AlfB1, LysC and Ctl24 were detected 24 h later. The data show the mean ± SD from three replicates. Statistical analysis was performed using the Students’ t test. *, 0.01 < p < 0.05; ***, 0.001 < p < 0.01. Each sample originated from five shrimp. (TIF) [file ppat.1010967.s008.tif]

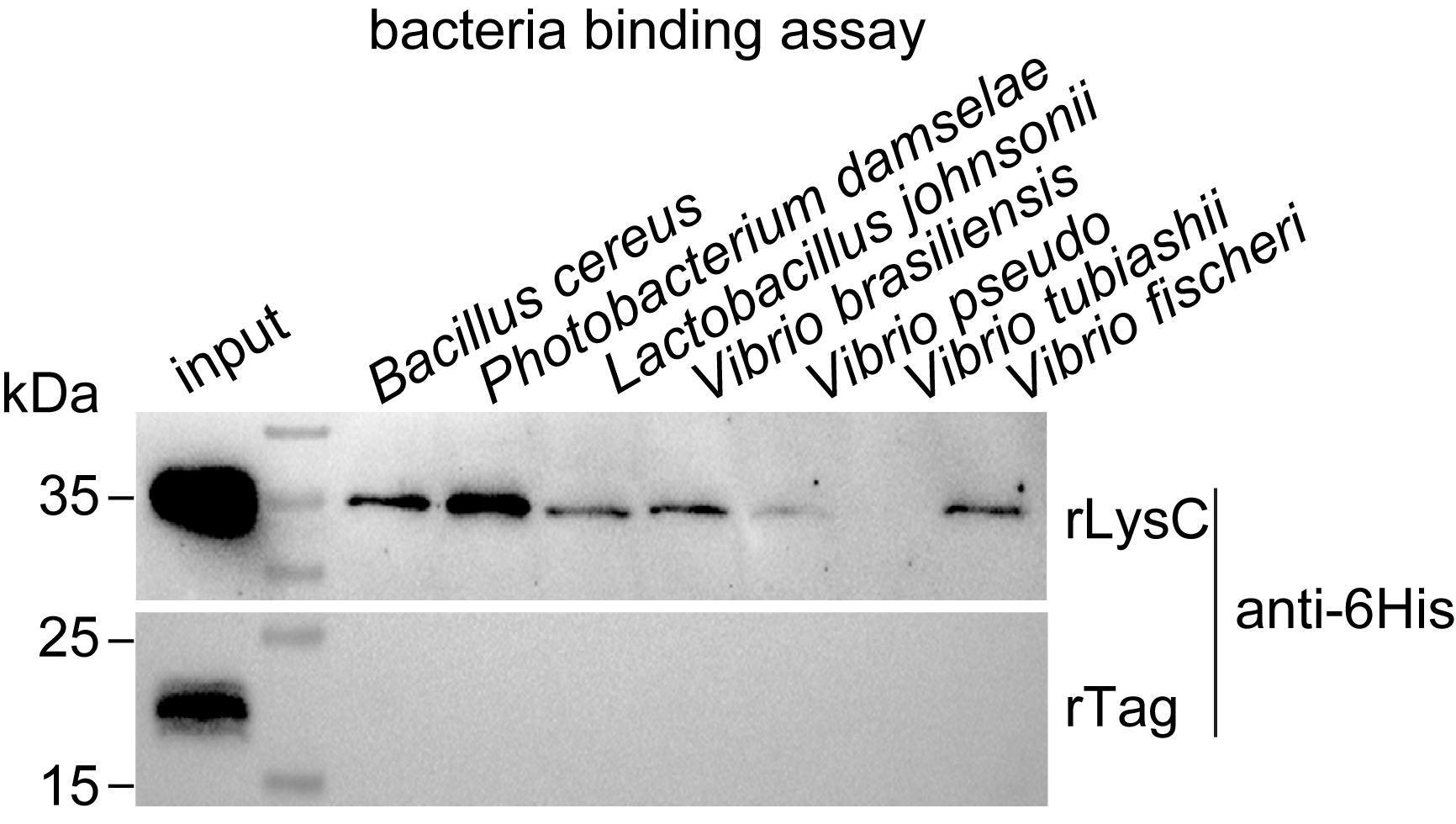

Supplement: S9 Fig — Bacteria (108 CFU) were incubated with rLysC or the control tag (50 μg/ml) for 2 h. After three washes with TBS, the bacterial pellets were processed, and the bound proteins were detected by western blotting using anti-6His antibodies. Data are representative of two repeats. (TIF) [file ppat.1010967.s009.tif]

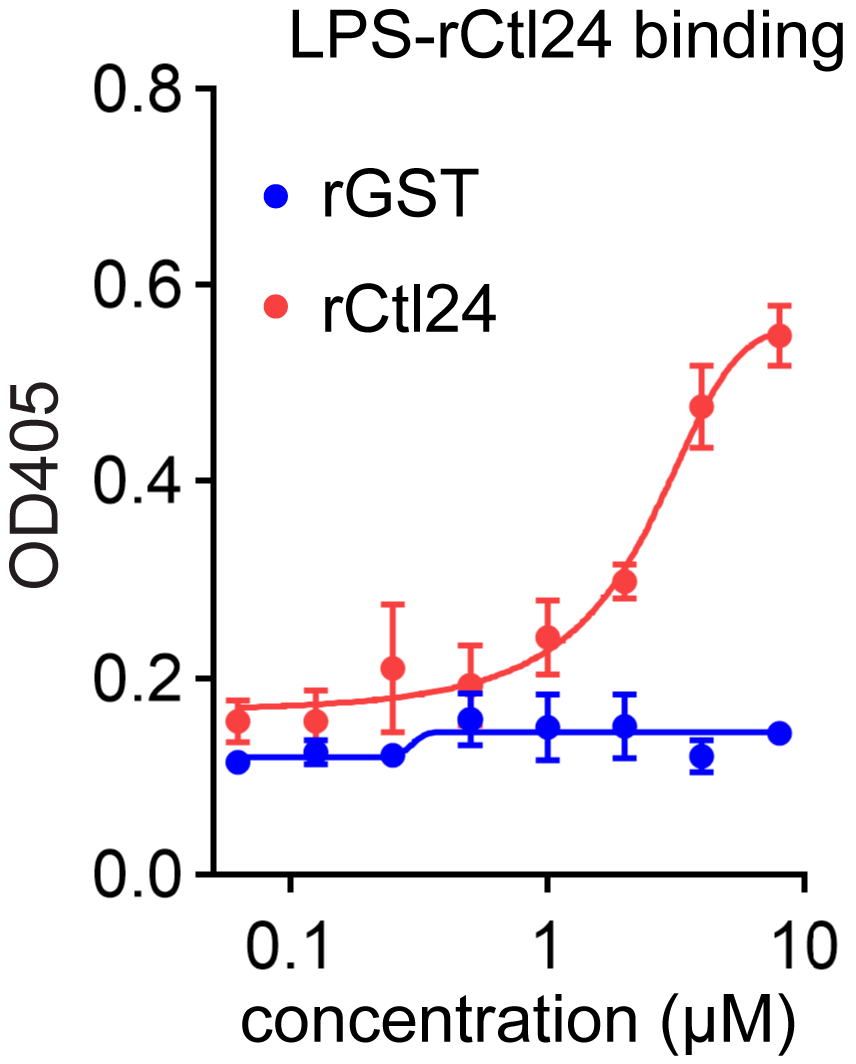

Supplement: S10 Fig — Gradient amount rLysC was added into 96-well plates pre-coated by LPS (L2880, Sigma-Aldrich; 4 μg/well), and the binding of rCTl24 was detected by an ELISA assay. The data show the mean ± SD from three replicates. (TIF) [file ppat.1010967.s010.tif]

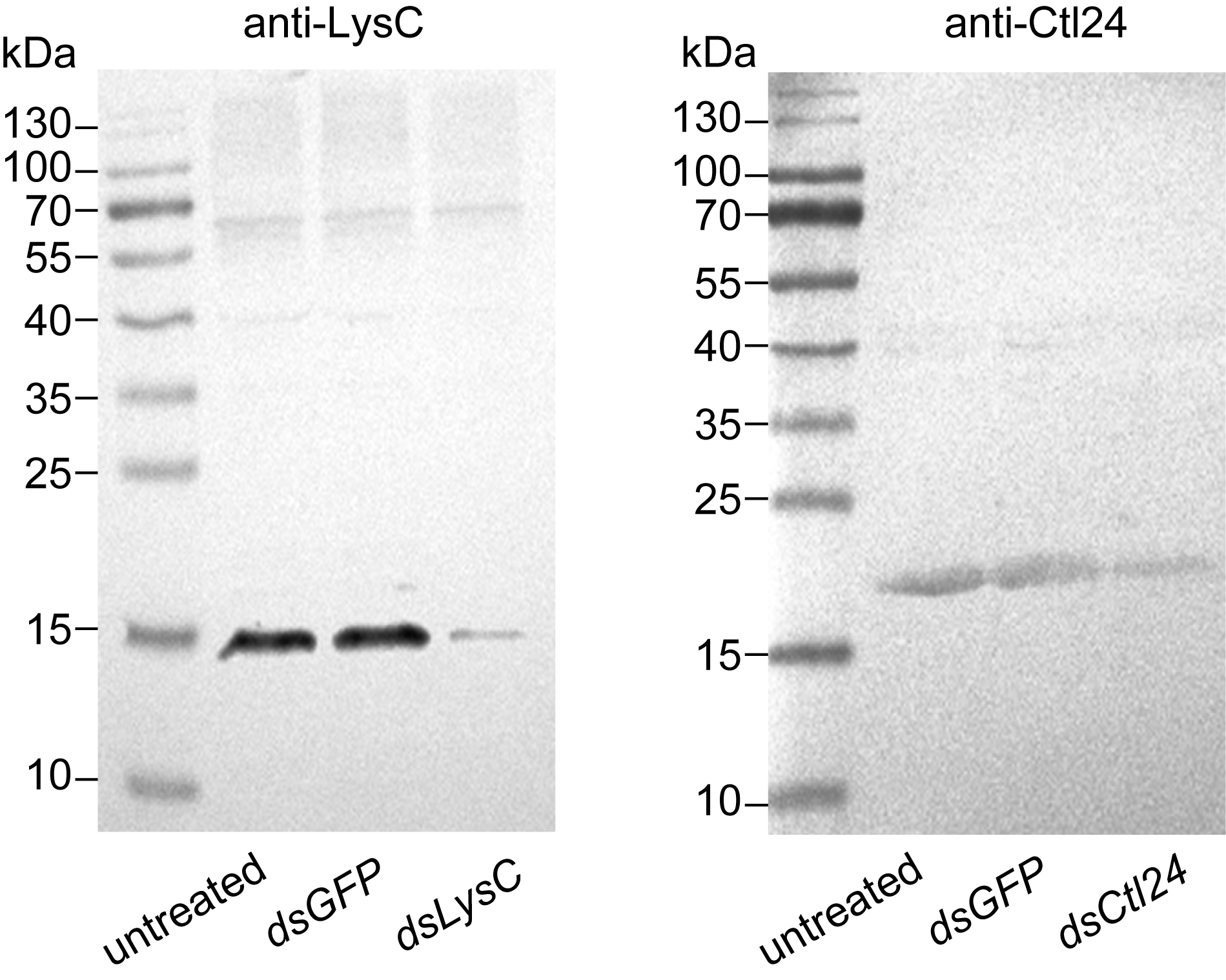

Supplement: S11 Fig — Intestine samples were collected 24 h after dsRNA application, and analyzed by western blotting using indicated antibodies. (TIF) [file ppat.1010967.s011.tif]
