# Supplementary material for: Small immune effectors coordinate peptidoglycan-derived immunity to regulate intestinal bacteria in shrimp
Source: PLoS Pathog. 2022 Nov 23;18(11):e1010967. doi: 10.1371/journal.ppat.1010967 (PMC9683584; doi:10.1371/journal.ppat.1010967)
Supplement: S2 Table — (DOCX) [file ppat.1010967.s013.docx]

**S2 Table. Primers and peptides used in this study**

| Primers | Sequence (5’-3’) |
| --- | --- |
| **RT-PCR** |  |
| LysCRTF | ATTTGCGGAGTTGCTGGAG |
| LysCRTR | GGAATTGGAAGGGCGAGAC |
| 16S rRNARTF | ACTCCTACGGGAGGCAGCAGT |
| 16S rRNARTR | TATTACCGCGGCTGCTGGC |
| *P. damselae*RTF | GGCTATTRCAKCAACSGAACACG |
| *P. damselae*RTR | TTTGGYGTTACRACTTGCACCCCT |
| AlfA1RTF | CTGGTCGGTTTCCTGGTGGC |
| AlfA1RTR | CCAACCTGGGCACCACATACTG |
| AlfB1RTF | CGGTGGTGGCCCTGGTGGCACTCTTCG |
| AlfB1RTR | GACTGGCTGCGTGTGCTGGCTTCCCCTC |
| AlfC1RTF | CGCTTCAAGGGTCGGATGTG |
| AlfC1RTR | CGAGCCTCTTCCTCCGTGATG |
| AlfC2RTF | TCCTGGTGGTGGCAGTGGCT |
| AlfC2RTR | TGCGGGTCTCGGCTTCTCCT |
| AlfD2RTF | CGCAGGCTTATGGAGGAC |
| AlfD2RTR | AGGTGACAGTGCCGAGGA |
| AlfE1RTF | TCCTAACCACGCAGTGCTTTGCTAATG |
| AlfE1RTR | GCTTTTCGGATTTGCCTTCGATGTTTG |
| AlfE2RTF | TGCCGTGTTCTCCTGCTTAT |
| AlfE2RTR | TTGGTGGGATTCGTGTGGT |
| Ctl4RTF | CCTGGAGGGCGAGTGTTTCTT |
| Ctl4RTR | GGAGCCACCCGTGTCTGATG |
| Ctl7RTF | AGGAAAATGCCAAAGTCAGGAGG |
| Ctl7RTR | CGAGACCAGTTGTAGATGCCACC |
| Ctl8RTF | GGGGACGACCAAGAGAACAG |
| Ctl8RTR | GGACAATGCCAAATCAAACAGT |
| Ctl9RTF | ATACAAGACCCTGGACGACTC |
| Ctl9RTR | ACCTCCTGGCTCCTAATGAA |
| Ctl12RTF | ACTCCTTCGCCGCCATCTT |
| Ctl12RTR | CATCCCTCGCTGGTTCTTCC |
| Ctl13RTF | CCGTCACCTTCCGCCACTACT |
| Ctl13RTR | TGAGGCTGGGCAGGGATAATAA |
| Ctl17RTF | TGTTTCAAGGCGGTGTTAG |
| Ctl17RTR | TTGGAGCAGTTGTTGTCGT |
| Ctl21RTF | GTGTGCTTGCCTTCCAGTCG |
| Ctl21RTR | GCAGTCCGTCAGTGTGTGTCC |
| Ctl23RTF | GGACGAGCGGCACGAAAG |
| Ctl23RTR | TTGAGGTGAAAGCAAGCGATGT |
| Ctl24RTF | TTTAGCCGTGTTCCGTG |
| Ctl24RTR | TCAAAACCATCCGACCC |
| Ctl26RTF | GCCAAGGTTTCCGCCGT |
| Ctl26RTR | CCCCGCCCAGAAGATGTAGTA |
| Ctl28RTF | CTTGCGAAGGTGCCGTG |
| Ctl28RTR | GGAGGGTTTCTGAAGGGGT |
| Ctl31RTF | GCGTCGGTGGTAAATGCT |
| Ctl31RTR | ATGGGAGTGTCGTCAATGG |
| Ctl33RTF | ACGGAGGAAGGTCCCAGAAC |
| Ctl33RTR | GAACAACAATAAAAAACCCAGGC |
| Ctl35RTF | ACACAAGCACCCATCACAAGC |
| Ctl35RTR | GAAGGTAAGGCAGTTTTCGTCG |
| Ctl36RTF | GCTGATGACCCGCCAAGA |
| Ctl36RTR | GCCTGAGAGCCACTCCCAA |
| Ctl46RTF | GGTGTGCTGTGCTTCGGG |
| Ctl46RTR | AGTCCATTTCCACTCGCCTT |
| Ctl52RTF | AAACATTTGGCTGGGTGGA |
| Ctl52RTR | TGACGAATCTCGGGGCA |
| Ctl53RTF | TGCGAGAAGCGAACAACG |
| Ctl53RTR | ACTCCACATCAAGCCGAAAA |
| Ctl56RTF | GTCCAACCTGTGGCTTCC |
| Ctl56RTR | CATCCTTTTTCTTCGCTTATC |
| Ctl59RTF | GTGCCCACGACCCAACTC |
| Ctl59RTR | AGGCTCAGCAGGAAAACGA |
| Ctl61RTF | CAACTCCCAGCCGATGT |
| Ctl61RTR | GCTCAGCAGGAAAACGAC |
| Ctl62RTF | CAGGCACCTCCTTTTGGG |
| Ctl62RTR | GGGACGAGTTCCTTTGGC |
| Ctl65RTF | TCCGCTGTGGATAGGCT |
| Ctl65RTR | GGTTCCGTGTGCTGGTT |
| Ctl66RTF | GCCAACGACTTCGCAGAG |
| Ctl66RTR | TGGAGACACAGAGGGTATTTTTC |
| Ctl71RTF | GGAAGGAAGACCTGGAAG |
| Ctl71RTR | AAAAATGAATGACAGCAAAA |
| Ctl72RTF | CGTTGTCCGAAGAAGCAC |
| Ctl72RTR | GGCGTCTCTATGGGTATTGTC |
| Ctl73RTF | GGGAGACGCTACATCATCA |
| Ctl73RTR | CCTCTTGGGCTGTGGTT |
| Ctl74RTF | CCTGCTGGACGAAAAATG |
| Ctl74RTR | ACACAAGTTGACCCCCG |
| Ctl75RTF | GACGCCAACGCATACGC |
| Ctl75RTR | CCAGCCGCAGGAGACATC |
| Ctl76RTF | GAAATGCCTTCTCGCCA |
| Ctl76RTR | CAACACGCTCATCTCTACTCG |
| Ctl78RTF | CTGCCCAGTTCGTTTCAC |
| Ctl78RTR | TCCACTTCCATTCCCCTT |
| Ctl85RTF | GCTTCACCTACGACGCAGA |
| Ctl85RTR | CACCCTTCCTCACAACCG |
| Ctl89RTF | AAGCCGAGGACTGGACG |
| Ctl89RTR | GCTGTTGTGCTCAGATGGA |
| β-actinRTF | CAGCCTTCCTTCCTGGGTATGG |
| β-actinRTR | GAGGGAGCGAGGGCAGTGATT |
| **RNAi** |  |
| LysCRNAiF | GCGTAATACGACTCACTATAGGTTTGCGGAGTTGCTGGAGA |
| LysCRNAiR | GCGTAATACGACTCACTATAGGGTTCAGAAATAGGACTGGGAC |
| AlfB1RNAiF | GCGTAATACGACTCACTATAGGCGTGTCTCCGCGTTGGTAA |
| AlfB1RNAiR | GCGTAATACGACTCACTATAGGACGAAGTCCCTGGCTGTCC |
| AlfD2RNAiF | GCGTAATACGACTCACTATAGGGATACCAGACCACGCATTT |
| AlfD2RNAiR | GCGTAATACGACTCACTATAGGGGTTTCGGATTCTCCCTTG |
| AlfE2RNAiF | GCGTAATACGACTCACTATAGGTCCTGCTTATGTCTGCTGC |
| AlfE2RNAiR | GCGTAATACGACTCACTATAGGCATTTCCGAGTTGGTGGG |
| Ctl13RNAiF | GCGTAATACGACTCACTATAGGCTACTGGACCAACACTTCGG |
| Ctl13RNAiR | GCGTAATACGACTCACTATAGGTGGGCAGGGATAATAAACA |
| Ctl24RNAiF | GCGTAATACGACTCACTATAGGGCCTTTACGAGGAGCATT |
| Ctl24RNAiR | GCGTAATACGACTCACTATAGGACAGAAGAGCCGAGCATC |
| Ctl36RNAiF | GCGTAATACGACTCACTATAGGTCCGTCCTTCTGATGCG |
| Ctl36RNAiR | GCGTAATACGACTCACTATAGGCCACTCCCAATGTCCCTC |
| Ctl46RNAiF | GCGTAATACGACTCACTATAGGCGTTCCAGGCAGTGGGTTT |
| Ctl46RNAiR | GCGTAATACGACTCACTATAGGTGCTTGTTGGGATTGAGGG |
| Ctl73RNAiF | GCGTAATACGACTCACTATAGGGTGCTTGAATGTGGTCGTC |
| Ctl73RNAiR | GCGTAATACGACTCACTATAGGGTAGCGTCTCCCGTGAACT |
| Ctl74RNAiF | GCGTAATACGACTCACTATAGGCAGTTGCCAGGTGTTTCTT |
| Ctl74RNAiR | GCGTAATACGACTCACTATAGGAGGGACGACTTTGCTATCT |
| Ctl75RNAiF | GCGTAATACGACTCACTATAGGATTTCTTTGGATGGTGGC |
| Ctl75RNAiR | GCGTAATACGACTCACTATAGGATCTGCGTATGCGTTGG |
| RelishRNAiF | GCGTAATACGACTCACTATAGGGTTATCGTCCTGTTTCCTTACC |
| RelishRNAiR | GCGTAATACGACTCACTATAGGGTGTTGGCATAGAGTCTTCCT |
| GFPRNAiF | GCGTAATACGACTCACTATAGGTGGTCCCAATTCTCGTGGAAC |
| GFPRNAiR | GCGTAATACGACTCACTATAGGCTTGAAGTTGACCTTGATGCC |
| **Recombinant expression** |  |
| LysCF | GCTGATATCGGATCCGAATTCAAGATTTTCAGGAAGTGTGA |
| LysCR | GTGGTGGTGGTGGTGCTCGAGCTAGAATGGGTAGATGGAATTG |
| Ctl24GSTF | GATCTGGTTCCGCGTGGATCCCTGGAGGGCAACAGTGTGGG |
| Ctl24GSTR | GTCACGATGCGGCCGCTCGAGCTAGATTTGACAGAGAGGCTTACAGTG |
| Peptide | Sequence |
| AlfB1 | CNFTVKPYIKKFQLYYKGRMWC |
